# Digital Peer Support to Increase Walking Among Older Adults: Cluster Randomized Trial

**DOI:** 10.2196/75708

**Published:** 2026-03-10

**Authors:** Atsushi Nakagomi, Noriyuki Abe, Takayuki Ueno, Gemmei Izuka, Yohei Kawasaki, Katsunori Kondo

**Affiliations:** 1 Department of Social Preventive Medical Sciences, Center for Preventive Medical Sciences, Chiba University, 1-33 Yayoicho, Inage-ku, Chiba, 263-8522, Japan, 81 432903176; 2 Research Team for Social Participation and Healthy Aging, Tokyo Metropolitan Institute for Geriatrics and Gerontology, Tokyo, Japan; 3 Department of Biostatistics, Graduate School of Medicine, Saitama Medical University, Saitama, Japan; 4 Research Department, Institute for Health Economics and Policy, Tokyo, Japan

**Keywords:** peer support, smartphone, digital health, physical activity, older adults, digital divide

## Abstract

**Background:**

As the population ages, older adults face an increasing risk of physical inactivity and related health complications, highlighting the need for scalable interventions. Smartphone-based programs have emerged as a promising strategy to support sustained physical activity among older adults.

**Objective:**

This study aimed to evaluate whether a smartphone lecture program incorporating a digital peer support app would increase physical activity among older adults, compared to a conventional smartphone lecture program.

**Methods:**

This 2-arm, 1:1 parallel-arm, cluster-randomized trial was conducted in 2 urban regions of Japan (Sumida Ward, Tokyo, and Chiba City, Chiba). Eligible participants were community-dwelling adults aged ≥60 years, able to walk independently, and smartphone users; exclusion criteria included prior use of the peer support app or medical restrictions on walking. Participants were recruited offline during community smartphone lectures (closed-group recruitment). The intervention combined face-to-face lectures with app-based peer support, while outcomes were assessed both objectively (via smartphones) and through self-administered paper questionnaires. All participants received a baseline smartphone lecture. Intervention participants attended 2 additional sessions using a digital peer support app (Minchalle; A10 Lab Inc), which included features such as daily step goals, peer sharing, and group encouragement. Control participants attended 2 standard follow-up smartphone lectures. The primary outcome was the change in weekly average daily step count from baseline to Week 12. Secondary outcomes included total metabolic equivalent of task (MET)–minutes per week (assessed via the International Physical Activity Questionnaire), walking time (≥30 minutes per day), daily smartphone use, and number of smartphone use purposes.

**Results:**

A total of 156 community-dwelling older adults were grouped into 40 clusters and randomized (20 intervention clusters, n=80 and 20 control clusters, n=76). In total, 124 participants (79.5%) completed the follow-up, and valid step data were available for 117 participants, with missing data ranging from 5.1% to 29.1%. Baseline daily steps averaged 3951 (SD 1686) in controls versus 4583 (SD 1973) in the intervention arm. An unadjusted mixed model for repeated measures showed significantly higher step changes for intervention participants at Week 12 (difference=579, 95% CI 36-1123; *P*=.04). No significant differences emerged for total METs (difference=646 MET-min per week, 95% CI –12 to 1303; *P*=.054) or walking ≥30 minutes per day (odds ratio [OR] 1.56, 95% CI 0.63-3.90; *P*=.33). However, the intervention arm demonstrated a significant increase in daily smartphone use (OR 4.10, 95% CI 1.15-14.6; *P*=.03) and in the number of smartphone use purposes (difference=0.58, 95% CI 0.12-1.05; *P*=.01).

**Conclusions:**

A smartphone lecture program integrated with app-based peer support led to modest but meaningful improvements in step counts among older Japanese adults, at Week 12 of the 12-week intervention. Future research should investigate long-term maintenance, additional measures of physical activity, and subpopulation responses to optimize digital health programs for older adults.

## Introduction

Physical activity is a key factor in healthy aging, helping prevent chronic diseases, maintain functional independence, and improve quality of life [1,2]. In Japan, where a rapidly aging population has raised concerns about inactivity and health care costs, the Ministry of Health, Labor, and Welfare (MHLW) has taken steps to address the issue. As part of the Healthy Japan 21 (3rd term) initiative, the MHLW set a target of 6000 steps per day for older adults by 2032, based on 2019 data showing averages of 5396 steps for men and 4656 for women [3]. The MHLW also released the Physical Activity and Exercise Guide for Health Promotion 2023 [4], emphasizing that even small increases in light-intensity activity yield significant health benefits, particularly for sedentary individuals [5]. As Japan’s older population continues to grow, scalable strategies to promote physical activity are an urgent public health need.

Evidence suggests behavior change strategies can promote sustained physical activity among older adults [6,7]. Intrapersonal strategies—such as action planning, goal setting, commitment, and self-monitoring—are commonly used in physical activity interventions [8,9]. Interpersonal strategies, especially peer support, are also gaining traction [10-12]. Social cognitive theory (SCT) provides a strong theoretical basis for these strategies, as it emphasizes the dynamic interaction between individual, behavioral, and social-environmental factors [13]. Key constructs of SCT include self-efficacy (confidence in one’s ability to maintain behavior), observational learning (acquiring new behaviors by observing others), and reinforcement (sustaining behaviors through social and environmental feedback). By drawing attention to how individual agency and social context interact, SCT offers a useful framework for understanding how both intrapersonal and interpersonal strategies can promote lasting engagement in physical activity among older adults.

With the growth of digital health technologies, many mobile apps now integrate behavior change techniques [14]. Alongside intrapersonal features, peer-support elements such as group challenges and shared goals are increasingly common [15-17]. For example, peer support can be structured in a smartphone app (Minchalle; A10 Lab Inc) through small groups of 3-5 participants who collaboratively select step goals, commit to posting updates at regular intervals, and encourage each other’s progress [17,18]. Integrating such peer support with intrapersonal strategies within the app has been shown to enhance user engagement and adherence to health goals [17,18].

However, older adults often face barriers—known as the “digital divide”—that hinder their use of technology-based interventions [19]. In Japan, local governments and community groups have introduced smartphone lectures to bridge this gap, yet sustained smartphone use remains a challenge. Digital peer support may offer a more lasting solution by providing ongoing group-based reinforcement, promoting both technology use and healthier behaviors. Still, few studies have compared smartphone lectures with and without digital peer support, highlighting a gap in empirical evidence.

This study evaluates whether adding digital peer support to smartphone lectures increases physical activity among older Japanese adults. Using a cluster-randomized controlled trial, we compared a conventional smartphone lecture to one combined with a peer support app grounded in behavioral science. A cluster design was required because the intervention was implemented within peer groups of 3-5 participants; randomizing individuals within the same group would have compromised the integrity of the peer support mechanism and risked contamination between trial arms. The primary objective of the trial was to evaluate changes in daily step counts at the individual participant level, while randomization occurred at the cluster (group) level to preserve the integrity of the peer support intervention. Participants were recruited in community settings and randomly assigned to either group. This design allowed us to examine whether ongoing digital peer support enhances motivation, sustains smartphone engagement, and improves physical activity outcomes.

## Methods

### Overview

The study was conducted in 2 urban municipalities within the Tokyo metropolitan region, Sumida Ward (Tokyo) and Chiba City (Chiba Prefecture). The trial was first implemented in Sumida Ward from September to December 2023 in collaboration with the municipal government and subsequently replicated in Chiba City from June to September 2024 through collaborations with local senior clubs. Although implemented in different municipalities, both settings are urban communities located near central Tokyo, allowing the study procedures to be applied consistently. This manuscript follows the 2010 CONSORT (Consolidated Standards of Reporting Trials) guidelines for cluster randomized trials (see Table S1 in Multimedia Appendix 1) [20] and the CONSORT-EHEALTH (Consolidated Standards of Reporting Trials of Electronic and Mobile Health Applications and Online Telehealth) guidelines (Checklist 1) [21].

### Design

This was a 2-arm, 1:1 parallel-arm, cluster-randomized trial that included a 2-week baseline measurement period followed by a 12-week intervention period. The unit of randomization (the cluster) was a peer support group consisting of 3-5 participants. In the intervention arm, these clusters functioned as peer groups within the Minchalle app, while in the control arm, participants were grouped to maintain comparability but did not engage in peer support during the trial. Control participants were informed that they would be offered the opportunity to form peer support groups after the trial (waiting-list design). Randomization at the cluster level was required to preserve the integrity of arm allocation.

### Participants and Recruitment

Recruitment took place from September 2023 to June 2024, in partnership with the Sumida Ward local government and senior clubs in Chiba City. Individuals were recruited in community venues during smartphone lectures hosted by the local municipal government or senior clubs, which aimed to bridge the digital divide and promote physical activity to prevent frailty among older adults. The local government and senior clubs outsourced lecture services to A10 Lab Inc., developer of the “Minchalle” app (Challenge with All), and Active Senior Outreach Nonprofit Organization, a nonprofit organization specializing in smartphone lectures for older adults.

These smartphone lectures were implemented as part of the annual programs conducted by the municipalities and senior clubs and were held in public community venues, typically accommodating 15‐30 older adults per session. Participants were approached in person at the beginning of the first session. Researchers informed all attendees about the study using slides and printed information sheets. Attendees were eligible to participate regardless of prior interest, and every individual attending the session was approached, ensuring a consistent and nonselective recruitment process.

Participants were eligible if they (1) were ≥60 years old, (2) could communicate in Japanese, (3) could read and provide informed consent, and (4) owned a smartphone at the time of consent. Participants were excluded if they (1) had any condition making participation infeasible (eg, inability to walk with a smartphone), (2) had a medical restriction on walking (eg, per doctor’s advice), or (3) had already used the Minchalle app.

Interested individuals were then screened by the research team based on the exclusion criteria. Those who met all criteria received a detailed explanation of the study procedures and provided written informed consent on-site. No participant was recruited through individual referrals, advertisements, or convenience sampling outside of the lecture setting.

Clusters were formed by grouping 3-5 participants who attended the same lecture session. No eligibility criteria were applied at the cluster level, and only individuals who provided informed consent were included. Individuals who did not meet the inclusion criteria or declined to participate were allowed to attend the lecture activities but were not included in the study cohort.

### Initial Session and Assessment

**Figure 1.**
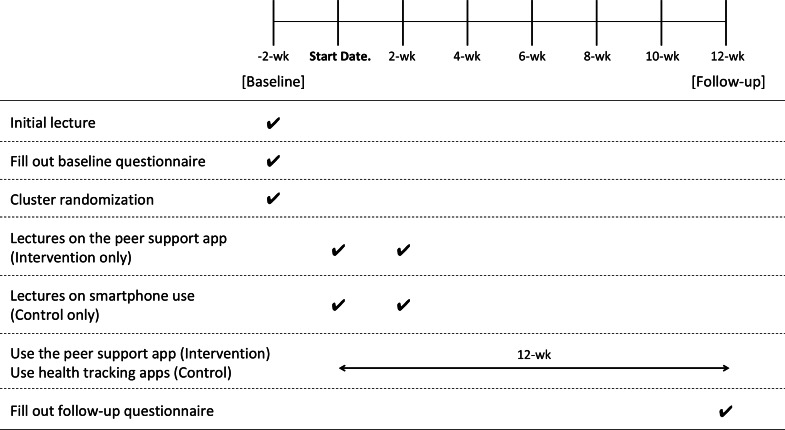
Timeline of study procedures.

Figure 1 outlines the study procedures. Consenting participants were organized into groups of 3‐5 and attended a smartphone lecture, followed by completion of baseline questionnaires. The lecture included a 15-minute presentation by a medical doctor on the health benefits of walking and a one-hour training session on installing and using health-tracking apps—Google Fit (Google LLC; Android) and Apple Health app (Apple Inc; iOS). Participants were encouraged to use basic app features, such as step counting and daily goal setting, incorporating intrapersonal behavior change strategies (action planning, goal setting, commitment, and self-monitoring). Participants were instructed to carry their smartphones daily to ensure continuous step count recording.

Before leaving the session, research staff verified successful installation of the app and confirmed that step-count tracking was active. Baseline paper questionnaires were completed on-site and collected immediately by trained staff. Baseline questionnaire data were linked to each participant using a deidentified study ID.

### Follow-Up Data Collection

At the 12-week follow-up session, participants completed follow-up paper questionnaires. During this session, research staff guided participants through the in-app procedures required to export and send their accumulated step count data to the Minchalle server using each participant’s deidentified study ID. Only participants who attended the follow-up session and completed these in-app actions were able to transmit their step data. The app provider then securely transferred the deidentified step-count dataset to the research team, where it was merged with questionnaire responses using the study ID. No personally identifiable information was shared, and all data were stored on secure, access-restricted servers.

### Randomization

Following the initial session and baseline assessment, randomization was conducted at the cluster level, with each group of 3-5 participants serving as the unit of allocation. The random allocation sequence was generated electronically using a random number generator with block sizes of 2-4 clusters. An independent allocation officer from the Institute for Assistance of Academic and Education implemented the sequence. The allocation sequence and block size were concealed from study personnel during enrollment, and arm assignments were revealed only after enrollment was finalized.

Individual participants were enrolled during community smartphone lectures and grouped pragmatically into clusters of 3-5 based on attendance at the same session. Randomization was therefore performed at the cluster level, while allocation concealment and consent were handled at the individual participant level.

Control arm participants were informed they would receive the intervention content after the trial (ie, a waiting-list control design).

### Blinding

Because of the nature of the intervention, participants and local staff could not be blinded to arm assignment. However, outcome assessment was based on step count data automatically recorded by participants’ smartphones, and data analysts were blinded to allocation, reducing the risk of bias.

The 2 study arms differed in that only the intervention arm received access to the Minchalle app and related training. Therefore, the interventions could not be made similar in appearance or content.

### Intervention

The intervention was a smartphone lecture program incorporating the digital peer support app Minchalle. All smartphone lecture programs were conducted in community venues in Sumida Ward (Tokyo) and Chiba City (Chiba Prefecture), which are typical venues for older adult smartphone lectures. The app, developed by A10 Lab Inc. and launched in 2015, was provided free of charge. Illustrations of the app are shown in Figure S1 in Multimedia Appendix 1. A structured description of the intervention is also provided according to the Template for Intervention Description and Replication (TIDieR) checklist (see Table S2 in Multimedia Appendix 1).

Minchalle integrates several behavior change techniques (BCTs) as defined by the BCT Taxonomy v1 [22]. The core element of Minchalle was the peer support component, in which groups of 3-5 members supported one another to achieve their goals by posting at least one photo and one message daily in the group chat (social support: general [BCT 3.1]). In addition, groups of 3-5 members selected daily step goals of 2500, 5000, or 10,000 steps (goal setting [BCT 1.1] and action planning [BCT 1.4]). Groups also selected a posting interval (every 4, 8, or 15 days). Members who failed to post within the agreed interval were removed (with the option to rejoin), reinforcing commitment (BCT 1.9: commitment). Step counts were automatically tracked and displayed in the chat, enabling self-monitoring of behavior (BCT 2.3) and promoting feedback and social comparison (BCT 6.2, 6.3) through the visibility of group achievements. This design was grounded in SCT, where peer encouragement, observational learning, and reinforcement are expected to enhance self-efficacy and sustain physical activity.

Two weeks after the initial baseline session, participants in the intervention arm attended 2 additional weekly lectures led by A10 Lab Inc, the developer of Minchalle. In these lectures, participants were instructed on how to install the app and configure initial settings, including selecting group step goals and defining posting intervals, after which members who failed to post within the interval would be removed. They also learned how to take photos, type messages, and post them in the group chat, supported by manuals provided during the sessions.

The intervention was delivered primarily at the cluster level (peer support groups of 3-5 participants), but individual participants used the app within their assigned peer support groups.

### Control

Participants in the control arm also attended 2 smartphone lectures, akin to typical sessions for older adults in Japan. These lectures covered basic smartphone functions, including email, web browsing, use of the messenger app LINE, and official local government apps or accounts. As in the initial lecture, they were encouraged to continue using Google Fit or Apple Health for step tracking. The final assessment for the control arm was conducted 3 months later, simultaneously with the intervention arm but in different locations.

### Outcomes

The primary outcome was the change in the weekly average of daily step counts from baseline to Week 12 (3 months after installation of the peer support app). After the baseline assessment, participants were asked to carry their smartphones at all times to capture daily steps. During the 2-week baseline period leading up to the second lecture, baseline step counts were measured. To minimize mismeasurement, any day with fewer than 1000 steps was treated as missing [23]. No changes to the prespecified primary outcome were made after trial commencement. All primary outcome data pertained to the individual participant level, although randomization was conducted at the cluster level.

Secondary outcomes included weekly step counts at other intervention weeks (Weeks 1‐11) and monthly averages (Months 1‐3), total physical activity (metabolic equivalent of task [MET] assessed by the International Physical Activity Questionnaire [IPAQ] short form) [24], self-reported walking time (≥30 minutes per day), daily smartphone use, and the number of smartphone use purposes at follow-up. The IPAQ short form was administered at baseline and follow-up to assess frequency and duration of vigorous, moderate, and walking activities (≥10 min bouts). Walking time was dichotomized as <30 or ≥30 minutes per day. Smartphone use was dichotomized as daily (almost daily) versus not daily. Smartphone use purposes were assessed from a checklist and summed to yield a total score from information search and browsing, work-related tasks, communication (eg, email, LINE, or video calls), taking photos or videos, using maps or traffic information, online banking, shopping, social media (eg, Facebook [Meta], Twitter [subsequently rebranded X; X Corp]), health apps (eg, step counters), entertainment (eg, video streaming, e-books, or music), gaming, and other uses.

### Statistical Analysis

The sample size was calculated assuming a 1-tailed test based on preliminary usage data with the same app; however, all subsequent analyses were conducted using 2-tailed tests at the 0.05 significance level. From pilot data provided by the app developer, we anticipated a medium effect size (Cohen *d*≈0.52) for changes in step counts. With *α*=.05, an intraclass correlation coefficient of 0.05 to account for clustering (mean cluster size of 4), and an expected attrition rate of 30%, we estimated that a total of 200 participants (100 per arm) would provide 80% power to detect a significant effect.

Baseline characteristics were summarized by treatment arm. Continuous variables were reported as mean (SD) and compared via 2-tailed 2‐sample *t* tests, while categorical variables were shown as frequencies (percentages) and analyzed via chi‐square or Fisher exact tests, using the *CreateTableOne* package in R (R Core Team).

Days with <1000 steps and other missing data were imputed; based on evidence, these often reflect incomplete capture [23,25]. Multiple imputation was performed using the package in R, incorporating baseline covariates and primary and secondary outcomes. Ten imputed datasets were generated, and estimates were combined using Rubin rules.

Separate imputation models were created for step count data (n=117) and questionnaire-based outcomes (n=124).

The primary analysis estimated the between-arm difference in the preregistered primary outcome—the change in weekly average daily step count from baseline to Week 12—using an unadjusted mixed model for repeated measures (MMRM). This model included treatment arm, week (categorical: baseline and Weeks 1‐12), and their interaction, with an unstructured covariance matrix at the participant level and cluster-robust standard errors to account for clustering of participants within groups.

Several sensitivity analyses were conducted to examine the robustness of the findings. These included (1) adjusted MMRM, (2) unadjusted, and (3) adjusted linear mixed-effects models with random intercepts at the participant and group levels, (4) unadjusted and (5) adjusted ANCOVA models with the change from baseline to Week 12 as the dependent variable, and (6) unadjusted MMRM using unimputed data, excluding days with missing or <1000 steps. For adjusted models, baseline covariates were selected a priori based on theoretical relevance and prior literature. Operating system (Android or iOS), study field (Sumida or Chiba), gender (men or women), education (high school or less vs college or more), marital status (married or unmarried), baseline smartphone use (daily vs not daily), and walking ≥30 minutes per day were treated as categorical variables. Age, self-rated health (0‐10 scale), and total MET-minutes per week were treated as continuous variables and modeled linearly.

Analyses of weekly step counts for Weeks 1‐11 and monthly averages (Months 1‐3) were conducted as secondary outcomes using the same unadjusted MMRM framework as the primary analysis. These analyses were not adjusted for multiple comparisons and were not part of the primary estimand, but are included to aid interpretation of temporal patterns.

Secondary outcomes obtained from the Week-12 follow-up questionnaire were analyzed using unadjusted models to maintain consistency with the primary analytic approach. Total physical activity (MET-minutes per week) and the number of smartphone use purposes were examined using ANCOVA, adjusting only for the baseline value of each outcome. Walking ≥30 minutes per day and daily smartphone use were analyzed using logistic regression with the same minimal adjustment.

Because a baseline age imbalance between arms was observed, we conducted an exploratory subgroup analysis stratified at the median age of 80 years. For each age stratum, the primary outcome was re-estimated using the unadjusted MMRM model described above. An interaction model including treatment × week × age (binary: ≥80 vs <80 years) was further examined to assess whether age modified the intervention effect.

### Ethical Considerations

This study received ethical approval from the Chiba University Ethical Committee (protocol number: M10626) and was registered with the University Hospital Medical Information Network (UMIN000051904). Written informed consent was obtained from all participants before randomization. All procedures adhered to the principles of the Declaration of Helsinki. Participant privacy and confidentiality were protected by deidentifying all data prior to analysis. Data were stored securely on password-protected servers accessible only to authorized study personnel. Only aggregated results are reported in this manuscript. Participants did not receive monetary compensation; however, they were allowed to use the smartphone app provided as part of the intervention, and no additional incentives were offered.

## Results

### Overview

Recruitment ended when the smartphone lecture series provided by the municipality and senior clubs—our sole recruitment venue—was completed, making additional enrollment infeasible. At that point, 177 participants had been enrolled. The analysis was conducted at this stage because recruitment had necessarily concluded, although the final sample size did not reach the originally planned statistical power.

**Figure 2.**
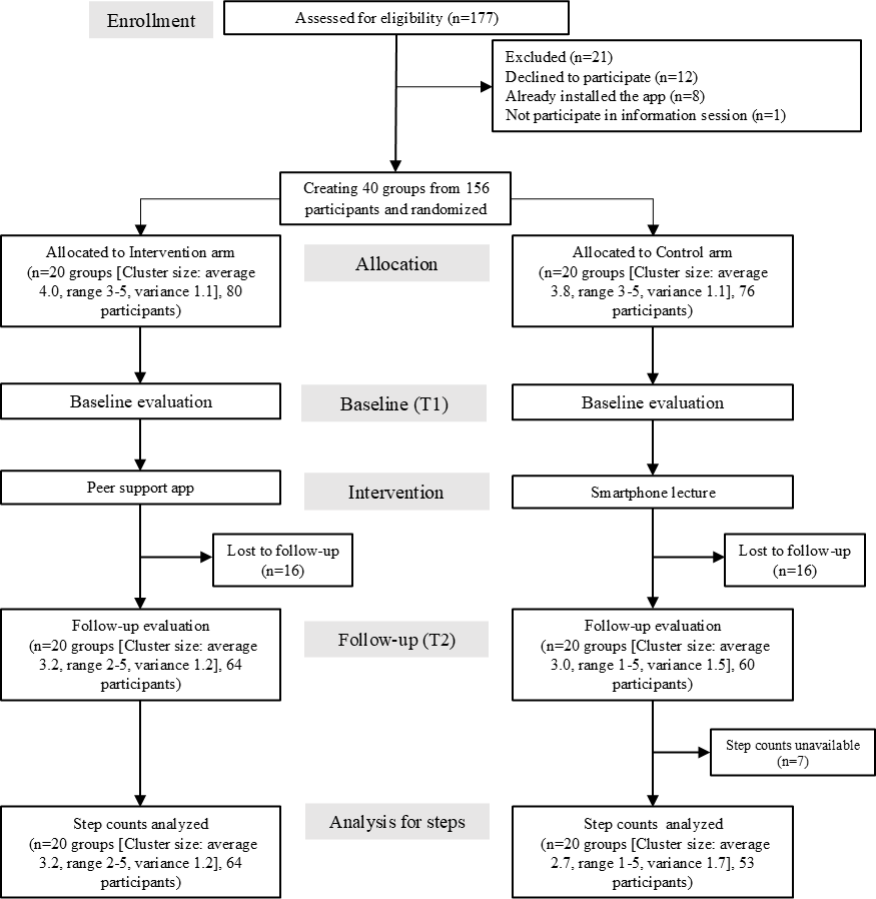
CONSORT (Consolidated Standards of Reporting Trials) flow diagram for study clusters and participants.

At the analysis, 40 groups comprising 156 participants were allocated either to an intervention arm (n=80, 20 groups) or a control arm (n=76, 20 groups; see Figure 2). Of the 156 enrolled participants, 124 (79.5%) completed the 3-month follow-up evaluation (64 in the intervention arm and 60 in the control arm), with step-count data successfully linked for 117 participants.

Missing step data ranged from 5.1% to 29.1%. As shown in Table 1, baseline characteristics were generally well balanced between arms, except for a significant difference in mean age (*P*=.009). Mean (SD) baseline daily steps were 3951 (1686) for the control arm and 4583 (1976) for the intervention arm.

**Table 1.** Characteristics of study participants.

Variables	Control (n=53)	Intervention (n=64)
Age (year), mean (SD)	81.2 (4.7)	79.0 (4.5)
Gender (Men), n (%)	17 (32.1)	18 (28.1)
OS (iOS), n (%)	9 (17.0)	18 (28.1)
Daily use of smartphone, n (%)	41 (77.4)	55 (85.9)
Education (college or more), n (%)	25 (47.2)	20 (31.7)
Married, n (%)	26 (49.1)	31 (48.4)
Self-rated health, mean (SD)	6.8 (2.0)	6.8 (1.9)
Total MET^a^, mean (SD)	3274 (1850)	3372 (2373)
Walking more than 30 minutes, n (%)	37 (69.8)	43 (67.2)
Baseline weekly average steps, mean (SD)	3951 (1686)	4583 (1976)

a MET: metabolic equivalent of task.

### Primary Outcome

**Figure 3.**
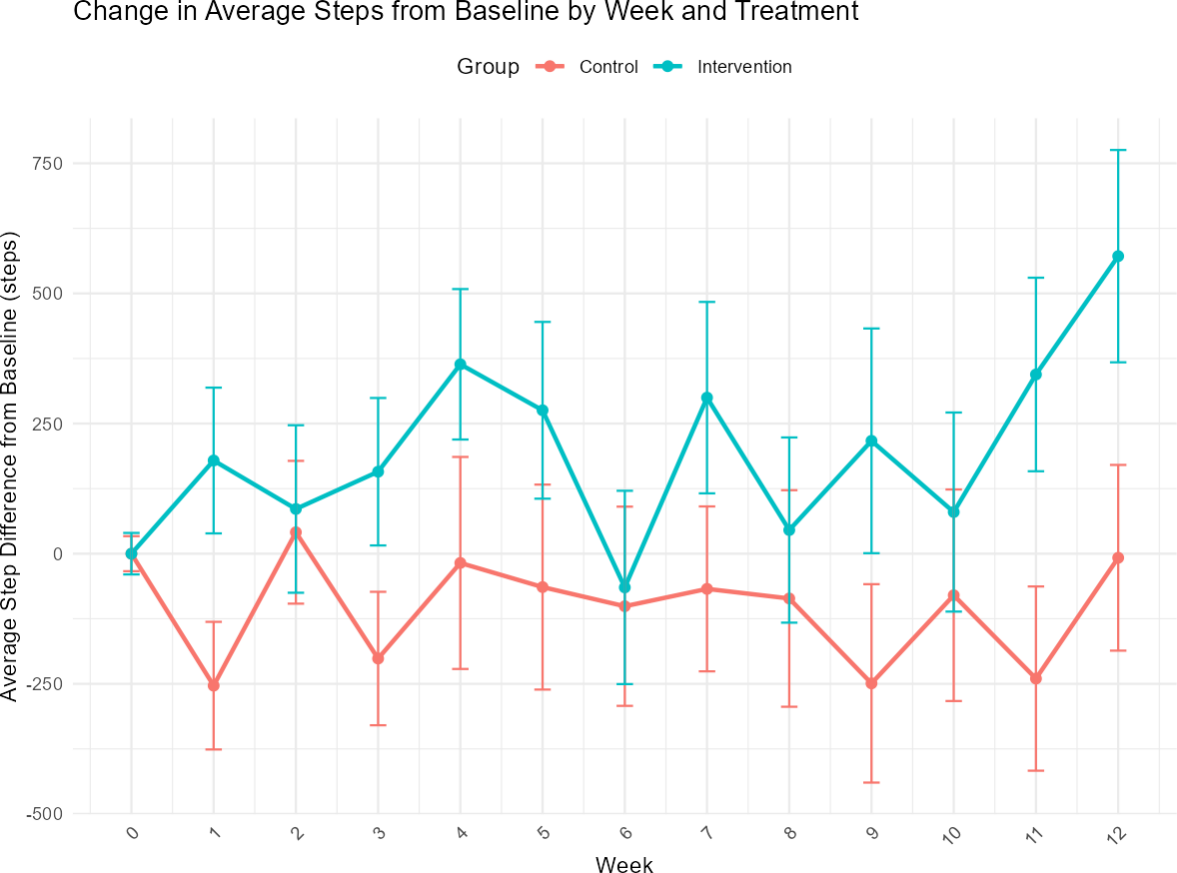
Unadjusted differences in mean daily steps by week and study arm.

The unadjusted differences in mean daily steps by week and study arm are illustrated in Figure 3. Across most time points, the intervention arm recorded higher step counts than the control.

The primary outcome was the change in average daily steps at week 12 compared with baseline. In the unadjusted MMRM model, the intervention arm recorded significantly higher steps than the control arm at Week 12 (difference=579 steps, 95% CI 36-1123**;** *P*=.04) (Table 2). The estimated cluster-level variance for the primary outcome was essentially zero (intraclass correlation coefficient ≈ 0.00), indicating minimal between-cluster variability after adjustment.

**Table 2.** Estimated between-arm differences in step counts by week (intervention minus control), unadjusted mixed-effects model for repeated measures.

Variables	Estimate^a^, *β* (95% CI)		*P* value
Intervention × Week 1^b^	433 (74 to 791)		.02
Intervention × Week 2	45 (−385 to 474)		.84
Intervention × Week 3	359 (−26 to 744)		.07
Intervention × Week 4	382 (−180 to 943)		.18
Intervention × Week 5	340 (−273 to 953)		.28
Intervention × Week 6	36 (−564 to 637)		.91
Intervention × Week 7	367 (−173 to 908)		.18
Intervention × Week 8	131 (−420 to 683)		.64
Intervention × Week 9	466 (−222 to 1154)		.18
Intervention × Week 10	160 (−434 to 754)		.60
Intervention × Week 11	584 (17 to 1151)		.04
Intervention × Week 12	579 (36 to 1123)		.04

a Estimates represent unadjusted between-arm differences at each week, derived from arm×week interaction terms.

b Intervention×week=product terms in the model.

The overall pattern remained consistent with the main analysis in sensitivity analysis: adjusted MMRM model (difference=539, 95% CI 92-985, *P*=.02); unadjusted mixed effects model (difference=589, 95% CI 110-1069; *P*=.02); adjusted mixed effects model (difference=526, 95% CI 65-987; *P*=.03); unadjusted ANCOVA model (difference=589, 95% CI 76-1101; *P*=.02); adjusted ANCOVA model (difference=489, 95% CI 33-946; *P*=.04); unadjusted MMRM model with complete case analysis (difference=1295, 95% CI 107-2483; *P*=.03).

### Secondary Outcomes

Exploratory analyses of weekly step counts (Weeks 1‐11) showed that the intervention arm had higher steps at Week 1 and Week 11 (Week 1: difference=433, 95% CI 74-791; *P*=.02; Week 11: difference=584, 95% CI 17-1151; *P*=.04, respectively) (Table 2). In a further exploratory analysis aggregating daily steps by month, the intervention arm showed greater, but not significant, differences at each month (Table S3 in Multimedia Appendix 1).

No significant arm differences were observed for physical activity measured by total METs or for self-reported walking time (≥30 minutes per day). The unadjusted difference in total METs was 646 MET-minutes per week (95% CI −12 to 1303; *P*=.054), and the odds ratio (OR) for walking ≥30 minutes per day was 1.56 (95% CI 0.63-3.90; *P*=.33; Table S4 in Multimedia Appendix 1). However, there was a suggestive trend toward an increase in daily smartphone use (OR 4.10, 95% CI 1.15-14.6; *P*=.03) and the number of smartphone use purposes (mean difference 0.58, 95% CI 0.12-1.05; *P*=.01) among participants in the intervention arm.

### Exploratory Post-Hoc Analyses

In exploratory subgroup analyses stratified by the median age of 80 years (younger: n=57; older: n=58), baseline age was well balanced within subgroups (younger: mean 76.2 vs 76.3 years; older: mean 84.2 vs 83.1 years). Intervention effects varied by age. Among older participants (≥80 years; n=58), significant treatment effects were observed, with between-arm differences exceeding 1000 steps at Week 12. In contrast, younger participants (<80 years; n=57) showed inconsistent and occasionally negative effects, with no sustained significant benefits. Consistent with these findings, the treatment × week × age interaction model indicated that intervention effects became stronger among participants aged 80 years or older in multiple weeks. Detailed estimates from the subgroup and interaction analyses are provided in Table S5 in Multimedia Appendix 1.

## Discussion

### Short Summary

This cluster-randomized controlled trial demonstrates that a smartphone lecture program, combined with a mobile app–based peer support component, may produce modest but meaningful increases in step counts among older adults in Japan compared to a conventional smartphone lecture program. The intervention arm recorded a statistically significant increase in average daily steps at Week 12 (the preregistered primary end point), with supportive differences observed at Weeks 1 and 11. All other weekly comparisons and monthly averages were exploratory and should be interpreted with caution, as no adjustment for multiple testing was applied. In addition, smartphone use and purposes were greater in the intervention arm. These results suggest that integrating peer support features into smartphone-based health initiatives could be a promising strategy for sustaining engagement and encouraging physical activity and smartphone use over time.

### Principal Findings

The peer support app appeared to stimulate progressive increases in step counts, particularly noticeable during the latter weeks. An important question is why intervention effects became most apparent in the latter part of the program (Weeks 11‐12). Novelty effects typically lead to high engagement at the outset of an intervention but tend to diminish over time, making it unlikely that novelty alone explains the late increase in steps. A more plausible explanation is that repeated goal setting, self-monitoring, and peer reinforcement gradually strengthened self-efficacy and supported the formation of walking habits, consistent with SCT. In this framework, the app’s design may have enhanced self-efficacy (through repeated goal attainment and encouragement), promoted observational learning (by making peers’ behaviors visible), and provided reinforcement (through accountability and supportive feedback).

In addition, seasonal and contextual factors may have contributed. The study was conducted in Sumida Ward, Tokyo, from September to December 2023. December includes cultural and social events that may have motivated participants to walk more and share photos with peers in the app. Also, in Chiba City, these were held from June to September 2024. Older adults might avoid walking outside in hot weather in July and August, while cooler weather in September may have encouraged outdoor activity. These contextual influences, alongside habit formation, may help explain why intervention effects were observed more strongly toward the end of the study period.

### Comparison to Prior Work

Our findings align with and extend prior evidence that interpersonal and peer-support components are particularly important for promoting and sustaining physical activity among older adults. McMahon et al [12] conducted a factorial randomized trial comparing intrapersonal and interpersonal behavior change strategies among community-dwelling adults aged ≥70 years and demonstrated that interventions including interpersonal components produced greater improvements in objectively measured physical activity, whereas intrapersonal components alone were less effective. Our results are consistent with this framework, while extending it to a pragmatic community education setting by embedding peer support within routinely delivered smartphone lectures rather than a research-intensive intervention.

Importantly, Tabira et al [17] evaluated the same digital peer-support smartphone app (Minchalle) used in our study in a nonrandomized controlled design among adults aged ≥65 years. They reported high feasibility, retention, and increases in physical activity, supporting the app’s acceptability and potential effectiveness in older populations. However, their study focused on feasibility and short-term behavioral change under a nonrandomized design, leaving uncertainty regarding causal effects. By contrast, our randomized controlled trial using the same app extends this prior work by strengthening causal inference and demonstrating effectiveness when Minchalle is implemented as part of existing smartphone lecture programs that are already widely disseminated across Japan.

In addition, Patel et al [18] showed in a large randomized trial that socially structured incentives embedded in behaviorally designed gamification can increase step counts, although the magnitude and persistence of effects varied by incentive type. While conducted primarily among working-age adults in a remote, workplace-adjacent context, their findings reinforce the broader principle that social and peer-based design features meaningfully influence physical activity behavior. Our study complements this literature by demonstrating that similar social mechanisms can be effectively operationalized in older adults using commercially available apps without requiring specialized devices or intensive program infrastructure.

#### Exploratory Post-Hoc Analyses

This study suggests that older participants (≥80 years) may derive greater benefit from app-based peer support, a group often most vulnerable to the digital divide [26]. By facilitating engagement with smartphones through peer support, our approach may help promote physical activity while also addressing inequities in digital participation. These findings are exploratory and not prespecified, and given the limited sample size, should be interpreted with caution.

### Secondary Findings

Despite the significant gains in step counts, we observed no significant arm differences in total METs or self-reported walking time (≥30 minutes per day). These discrepancies may stem from the inherently coarse nature of subjective physical activity measures, which can miss nuances that more objective step-tracking captures [12,27]. Indeed, correlations between self-report and objectively measured physical activity in older adults are positive, yet often moderate at best [28]. Future studies integrating more comprehensive, sensor-based activity assessments will help clarify whether peer support can robustly influence other domains of physical activity beyond daily step counts.

There was also a suggestive trend toward increased daily smartphone use in the intervention arm, implying that digital peer support may facilitate broader technology adoption. This is consistent with the primary aim of smartphone lectures for older adults and suggests that digital peer support apps can effectively enhance participants’ confidence and willingness to explore additional smartphone features.

### Limitations

Several limitations merit consideration. First, the final sample size was smaller than planned, which reduced statistical power, particularly for secondary outcomes. Attrition (≈20%) further reduced the analytic sample, mainly due to nonattendance at the 3-month follow-up lecture, and step-count data from 7 participants were lost because of a technical linkage error. Second, missing data and imputation procedures, while consistent with prior interventions, could introduce bias if data were not missing at random. Third, digital peer support may not uniformly appeal to all participants, potentially limiting generalizability. Future studies should examine heterogeneous responses to peer support for physical activity and smartphone adoption. Fourth, although we reported step count differences at multiple weekly and monthly time points, only the between-arm difference at Week 12 was prespecified as the confirmatory primary end point. All other weekly and monthly comparisons were exploratory and not adjusted for multiplicity; therefore, these findings should be interpreted with caution. Fifth, no formal arm sequential testing procedure (eg, alpha-spending function) was applied to adjust for multiple looks at the data. Recruitment was concluded after 177 participants had been enrolled, based on the completion of the smartphone lectures organized by the local municipal government and senior clubs. The absence of a formal stopping rule represents a limitation with respect to strict error rate control. Sixth, SCT constructs were not directly measured in this trial, and the hypothesized mechanisms (eg, self-efficacy, observational learning, and reinforcement) remain speculative. Seventh, no process measures such as outcome expectancies or social support perceptions were collected, and detailed app usage metrics (eg, posting volume, response rates, and network analyses) were not available, limiting the ability to fully evaluate the peer support component. Eighth, baseline age differed modestly between arms (79.0 years in intervention vs 81.2 years in control). However, exploratory analyses suggested that older participants benefited more from the intervention. Therefore, this imbalance would likely have biased estimates toward underestimation of the true effect rather than exaggeration. These age-stratified analyses were exploratory, not prespecified, and should be interpreted with caution given the limited sample size. Ninth, although the Minchalle app interface and features were identical on Android and iOS, potential platform-specific differences in step data capture (eg, Google Fit vs Apple Health) cannot be excluded. Due to the small number of iOS users (n=27), subgroup analyses by operating system were not feasible, and future studies should address this more explicitly. Tenth, individual-level attrition resulted in smaller cluster sizes at follow-up, particularly in the control arm, where some clusters ultimately had only one participant contributing data. Participants who did not attend the follow-up session were unable to provide questionnaire or step-count data, and in the intervention arm, we were also unable to monitor their continued use of the peer-support app. As a result, it remains uncertain whether any intervention clusters functionally shrank to very small active groups during the trial period. This uncertainty may influence the interpretation of peer-related effects, as reduced group size could weaken the social facilitation or mutual-accountability mechanisms central to the intervention. Finally, the external validity of our findings should be interpreted with caution. This trial was conducted in 2 urban areas of Japan, where walkable environments and access to digital infrastructure are relatively well developed. The results may not directly translate to rural areas with limited walkability or to contexts with lower smartphone and internet penetration. In addition, cultural differences in peer support and technology use may influence the effectiveness of similar interventions in international settings. These contextual factors should be carefully considered when applying our findings to broader populations.

### Conclusions

In conclusion, our results suggest that a mobile peer support intervention combined with a smartphone lecture can yield significant increases in step counts among older Japanese adults. While secondary measures of total physical activity and self-reported walking time did not show a comparable effect, the observed gains in daily steps underscore the potential of app-based peer support for promoting sustained walking behavior. Moreover, a marginally significant increase in daily smartphone use and a significant increase in the number of smartphone use purposes highlight the possibility that a digital peer support program can enhance technology adoption among older adults. Future research should explore longer-term maintenance and identify which segments of the older population stand to benefit the most in order to optimize digital health interventions for this rapidly growing demographic.

## Supplementary material

10.2196/75708 Multimedia Appendix 1 Supplementary figure and additional statistical analysis tables.

10.2196/75708 Checklist 1 CONSORT-eHEALTH checklist (V 1.6.1)

## Abbreviations

BCT: behavior change technique
CONSORT: Consolidated Standards of Reporting Trials
CONSORT-EHEALTH: Consolidated Standards of Reporting Trials of Electronic and Mobile Health Applications and Online Telehealth
IPAQ: International Physical Activity Questionnaire
MET: metabolic equivalent of task
MHLW: Ministry of Health, Labor, and Welfare
MMRM: mixed model for repeated measures
OR: odds ratio
SCT: social cognitive theory
TIDieR: Template for Intervention Description and Replication
