# Supplementary material for: Digital Peer Support to Increase Walking Among Older Adults: Cluster Randomized Trial
Source: J Med Internet Res. 2026 Mar 10;28:e75708. doi: 10.2196/75708 (PMC12974999; doi:10.2196/75708)
Supplement: Multimedia Appendix 1 [file jmir-v28-e75708-s001.docx]

Figure S1 Examples of app screens.

(A) Select a group. (B) Post a photo taken that day, step count, and comments on the group. (C) Posts are displayed in the group chat. The total number of group steps is displayed. (D) Response to posts by group members.


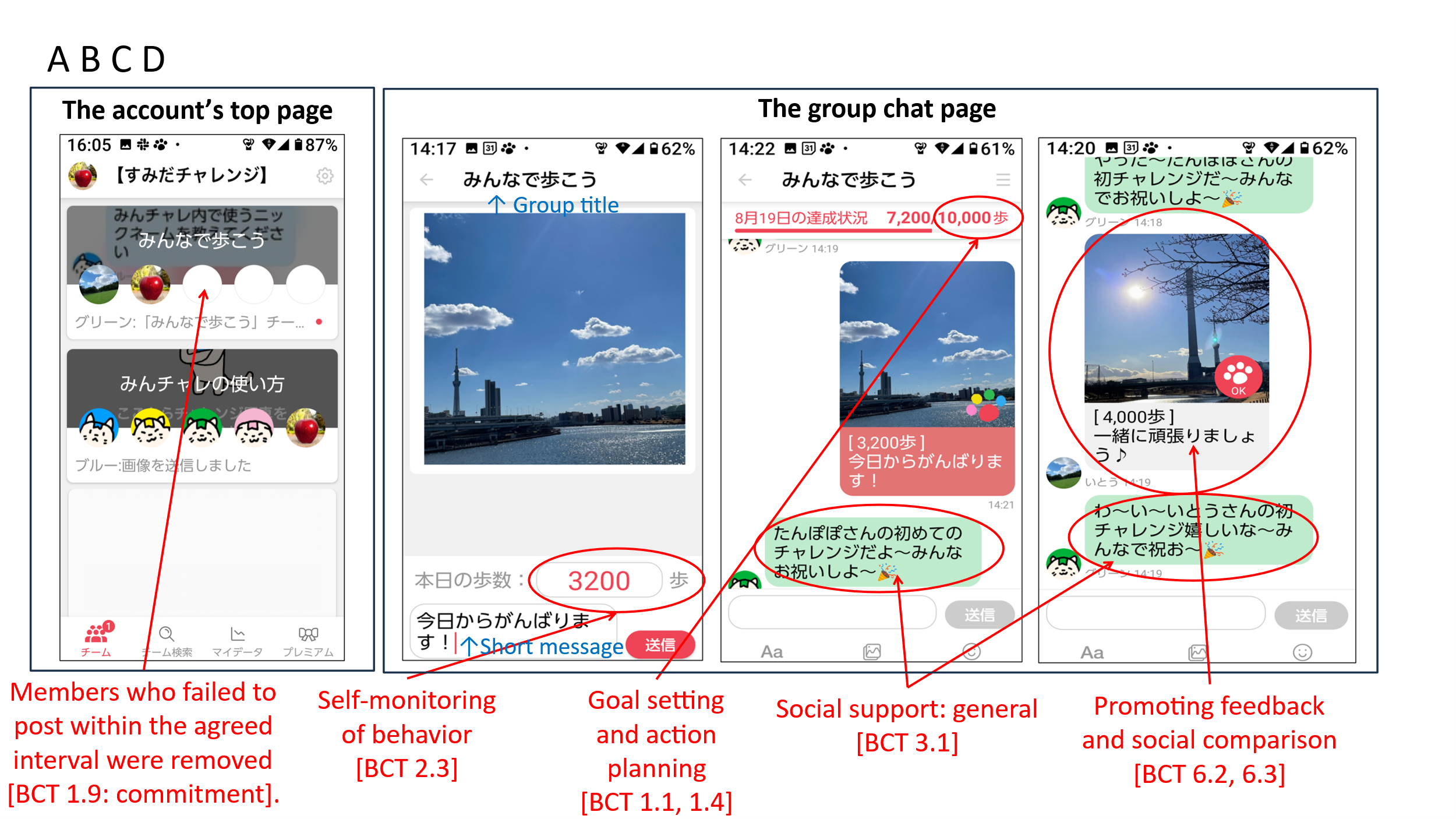


Table S1 CONSORT 2010 checklist of information to include when reporting a cluster randomized trial

| **Section/topic and item No** | **Standard checklist item** | **Extension for cluster designs** | **Page No*** |
| --- | --- | --- | --- |
| **Title and abstract** | | | |
| 1a | Identification as a randomised trial in the title | Identification as a cluster randomised trial in the title | 1 |
| 1b | Structured summary of trial design, methods, results, and conclusions (for specific guidance see CONSORT for abstracts)^11 12^ | See table 2 | 2 |
| **Introduction** | | | |
| Background and objectives: |  |  |  |
| 2a | Scientific background and explanation of rationale | Rationale for using a cluster design | 5 |
| 2b | Specific objectives or hypotheses | Whether objectives pertain to the cluster level, the individual participant level, or both | 5 |
| **Methods** | | | |
| Trial design: |  |  |  |
| 3a | Description of trial design (such as parallel, factorial) including allocation ratio | Definition of cluster and description of how the design features apply to the clusters | 5 |
| 3b | Important changes to methods after trial commencement (such as eligibility criteria), with reasons |  | NA |
| Participants: |  |  |  |
| 4a | Eligibility criteria for participants | Eligibility criteria for clusters | NA |
| 4b | Settings and locations where the data were collected |  | 5 |
| Interventions: |  |  |  |
| 5 | The interventions for each group with sufficient details to allow replication, including how and when they were actually administered | Whether interventions pertain to the cluster level, the individual participant level, or both | 7 |
| Outcomes: |  |  |  |
| 6a | Completely defined prespecified primary and secondary outcome measures, including how and when they were assessed | Whether outcome measures pertain to the cluster level, the individual participant level, or both | 8 |
| 6b | Any changes to trial outcomes after the trial commenced, with reasons |  | NA |
| Sample size: |  |  |  |
| 7a | How sample size was determined | Method of calculation, number of clusters(s) (and whether equal or unequal cluster sizes are assumed), cluster size, a coefficient of intracluster correlation (ICC or *k*), and an indication of its uncertainty | 8 |
| 7b | When applicable, explanation of any interim analyses and stopping guidelines |  | 9 |
| **Randomisation** | | | |
| Sequence generation: |  |  |  |
| 8a | Method used to generate the random allocation sequence |  | 6 |
| 8b | Type of randomisation; details of any restriction (such as blocking and block size) | Details of stratification or matching if used | 6 |
| Allocation concealment mechanism: |  |  |  |
| 9 | Mechanism used to implement the random allocation sequence (such as sequentially numbered containers), describing any steps taken to conceal the sequence until interventions were assigned | Specification that allocation was based on clusters rather than individuals and whether allocation concealment (if any) was at the cluster level, the individual participant level, or both | 6 |
| Implementation: |  |  |  |
| 10 | Who generated the random allocation sequence, who enrolled participants, and who assigned participants to interventions | Replaced by 10a, 10b, and 10c |  |
| 10a |  | Who generated the random allocation sequence, who enrolled clusters, and who assigned clusters to interventions | 6 |
| 10b |  | Mechanism by which individual participants were included in clusters for the purposes of the trial (such as complete enumeration, random sampling) | 6 |
| 10c |  | From whom consent was sought (representatives of the cluster, or individual cluster members, or both) and whether consent was sought before or after randomisation | 5 |
| Blinding: |  |  |  |
| 11a | If done, who was blinded after assignment to interventions (for example, participants, care providers, those assessing outcomes) and how |  | 5 |
| 11b | If relevant, description of the similarity of interventions |  | NA |
| Statistical methods: |  |  |  |
| 12a | Statistical methods used to compare groups for primary and secondary outcomes | How clustering was taken into account | 9 |
| 12b | Methods for additional analyses, such as subgroup analyses and adjusted analyses |  | 9 |
| **Results** | | | |
| Participant flow (a diagram is strongly recommended): |  |  |  |
| 13a | For each group, the numbers of participants who were randomly assigned, received intended treatment, and were analysed for the primary outcome | For each group, the numbers of clusters that were randomly assigned, received intended treatment, and were analysed for the primary outcome | Figure 2 |
| 13b | For each group, losses and exclusions after randomisation, together with reasons | For each group, losses and exclusions for both clusters and individual cluster members | Figure 2 |
| Recruitment: |  |  |  |
| 14a | Dates defining the periods of recruitment and follow-up |  | 10 |
| 14b | Why the trial ended or was stopped |  | 10 |
| Baseline data: |  |  |  |
| 15 | A table showing baseline demographic and clinical characteristics for each group | Baseline characteristics for the individual and cluster levels as applicable for each group | 10, Table 1 |
| Numbers analysed: |  |  |  |
| 16 | For each group, number of participants (denominator) included in each analysis and whether the analysis was by original assigned groups | For each group, number of clusters included in each analysis | Figure 2 |
| Outcomes and estimation: |  |  |  |
| 17a | For each primary and secondary outcome, results for each group, and the estimated effect size and its precision (such as 95% confidence interval) | Results at the individual or cluster level as applicable and a coefficient of intracluster correlation (ICC or *k*) for each primary outcome | 11, Table 2 |
| 17b | For binary outcomes, presentation of both absolute and relative effect sizes is recommended |  | NA |
| Ancillary analyses: |  |  |  |
| 18 | Results of any other analyses performed, including subgroup analyses and adjusted analyses, distinguishing prespecified from exploratory |  | Supplementary Table 3, 4, 5 |
| Harms: |  |  |  |
| 19 | All important harms or unintended effects in each group (for specific guidance see CONSORT for harms106) |  | NA |
| **Discussion** | | | |
| Limitations: |  |  |  |
| 20 | Trial limitations, addressing sources of potential bias, imprecision, and, if relevant, multiplicity of analyses |  | 14 |
| Generalisability: |  |  |  |
| 21 | Generalisability (external validity, applicability) of the trial findings | Generalisability to clusters and/or individual participants (as relevant) | 15 |
| Interpretation: |  |  |  |
| 22 | Interpretation consistent with results, balancing benefits and harms, and considering other relevant evidence |  | 15 |
| **Other information** | | | |
| Registration: |  |  |  |
| 23 | Registration number and name of trial registry |  | University Hospital Medical Information Network (UMIN000051904) |
| Protocol: |  |  |  |
| 24 | Where the full trial protocol can be accessed, if available |  | NA |
| Funding: |  |  |  |
| 25 | Sources of funding and other support (such as supply of drugs), role of funders |  | 16 |

Table S2 TIDieR Checklist for Smartphone Lecture Program Incorporating Digital Peer Support App

| **Item** | **Description** |
| --- | --- |
| 1. Brief Name | Smartphone lecture program incorporating digital peer support app (MinChalle) |
| 2. Why (Rationale, Theory, Goal) | The main content of the intervention was the peer support component. This was grounded in Social Cognitive Theory (SCT), in which peer encouragement, observational learning, and reinforcement are expected to enhance self-efficacy and sustain physical activity. Other behavior change techniques such as goal setting, action planning, and self-monitoring were also present in the MinChalle app and functioned as supportive elements. |
| 3. What: Materials | The intervention used the MinChalle app, which was provided free of charge, alongside standard smartphone health-tracking apps (Google Fit for Android, Healthcare for iOS). Supplementary educational materials included lecture slides and manuals for installing and using the apps. |
| 4. What: Procedures | All participants first attended a baseline lecture consisting of a 15-minute physician-led health presentation and one hour of training on basic app use. Intervention participants then attended two further lectures, which focused on installing and practicing the MinChalle app. Within small groups of three to five, members selected a daily step goal (2,500, 5,000, or 10,000 steps) and agreed on a posting interval (4, 8, or 15 days). Each member was encouraged to post at least one photo or message daily; those who failed to post within the agreed interval were temporarily removed from the group but could rejoin. Step counts were automatically tracked and shared in the group chat, enabling visibility of progress and promoting feedback, accountability, and social comparison. |
| 5. Who provided | The additional MinChalle sessions were delivered by A10 Lab Inc., the developer of the app. The initial baseline lecture included a health talk provided by a physician. Local governments and senior clubs supported the logistics of participant recruitment and lecture delivery. |
| 6. How | Delivery combined in-person, face-to-face smartphone lectures with group-based digital interaction through the MinChalle smartphone app. |
| 7. Where | The program was conducted in community venues in Sumida Ward (Tokyo) and Chiba City (Chiba Prefecture), which are typical locations for older adult smartphone lecture programs. |
| 8. When and how much | The intervention consisted of one baseline lecture, followed by two weekly MinChalle-focused lectures. After the training, app-based peer support continued daily for 12 weeks. |
| 9. Tailoring | Tailoring occurred through group selection of daily step goals (2,500, 5,000, or 10,000 steps) and posting intervals (every 4, 8, or 15 days). Members who missed posts within the agreed interval were temporarily removed, with the option to rejoin. |
| 10. Modifications | No modifications to the intervention were reported during the study. |
| 11. How well: Planned adherence/fidelity assessment | Participants were instructed to carry their smartphones daily to ensure continuous step count recording. Step data were automatically tracked by the app, and posting requirements served as built-in reinforcement. |
| 12. How well: Actual adherence/fidelity | Adherence to the intervention was not systematically monitored beyond step count data and attendance at follow-up lectures. No detailed usage metrics (such as frequency of posts or app log-ins) were collected. Approximately 79.5% of participants completed the three-month follow-up, and valid step data were obtained for 117 participants. Missing step data ranged from 5.1% to 29.1% of days and were addressed through imputation. |

Table S3 Estimated Between-Arm Differences in Step Counts by Months (Intervention minus Control), an unadjusted mixed model for repeated measures model

|  | Estimate | 95% CI | *P*-value |
| --- | --- | --- | --- |
| Intervention * Month 1 | 304 | (-65 to 674) | .11 |
| Intervention * Month 2 | 219 | (-314 to 751) | .42 |
| Intervention * Month 3 | 447 | (-87 to 981) | .10 |

Abbreviations:

CI: confidence interval

Table S4 Secondary outcomes from unadjusted models

|  | Estimate | 95% CI | *P*-value |
| --- | --- | --- | --- |
| Total MET | 646 | (-12 to 1303) | .054 |
| Walking* | 1.56 | (0.63 to 3.9) | .33 |
| Daily use* | 4.1 | (1.15 to 14.6) | .03 |
| Purposes | 0.58 | (0.12 to 1.05) | .01 |

Abbreviations:

CI: confidence interval; MET: metabolic equivalent.

*Odds ratio is shown. Other estimates are coefficients from linear models.

Table S5 Estimated Between-Arm Differences in Step Counts by Week (Intervention minus Control) among Younger (79 or younger, n = 57) and Older (80 or older, n = 58) Participants, an unadjusted mixed model for repeated measures model

|  | Younger (79 or younger, n = 57) | | |  | Older (80 or older, n = 58) | | |  |  |
| --- | --- | --- | --- | --- | --- | --- | --- | --- | --- |
|  | Estimate | 95% CI | P-value |  | Estimate | 95% CI | P-value |  | *P* for interaction |
| Intervention * Week 1 | 298 | (-259, 854) | .29 |  | 489 | (58, 921) | 0.03 |  | .56 |
| Intervention * Week 2 | -207 | (-729, 314) | .44 |  | 136 | (-443, 714) | 0.65 |  | .37 |
| Intervention * Week 3 | 12 | (-439, 462) | .96 |  | 563 | (84, 1041) | 0.02 |  | .07 |
| Intervention * Week 4 | -486 | (-1428, 456) | .31 |  | 923 | (354, 1493) | 0.00 |  | .01 |
| Intervention * Week 5 | -363 | (-1566, 839) | .55 |  | 756 | (301, 1211) | 0.00 |  | .07 |
| Intervention * Week 6 | -852 | (-1743, 39) | .06 |  | 642 | (29, 1255) | 0.04 |  | .002 |
| Intervention * Week 7 | -400 | (-1174, 373) | .31 |  | 835 | (312, 1359) | 0.00 |  | .002 |
| Intervention * Week 8 | -664 | (-1537, 210) | .14 |  | 584 | (64, 1103) | 0.03 |  | .01 |
| Intervention * Week 9 | -385 | (-1345, 574) | .43 |  | 981 | (279, 1684) | 0.01 |  | .009 |
| Intervention * Week 10 | -957 | (-2043, 128) | .08 |  | 884 | (318, 1449) | 0.00 |  | .004 |
| Intervention * Week 11 | 25 | (-935, 985) | .96 |  | 894 | (304, 1485) | 0.00 |  | .10 |
| Intervention * Week 12 | -356 | (-1247, 534) | .43 |  | 1198 | (624, 1771) | 0.00 |  | .003 |

CI: confidence interval.
