# Supplementary material for: Digital Peer Support to Increase Walking Among Older Adults: Cluster Randomized Trial
Source: J Med Internet Res. 2026 Mar 10;28:e75708. doi: 10.2196/75708 (PMC12974999; doi:10.2196/75708)
Supplement: Checklist 1 [file jmir-v28-e75708-s002.pdf]

CONSORT-EHEALTH (V 1.6.1) - Submission/Publication Form

The CONSORT-EHEALTH checklist is intended for authors of randomized trials evaluating web-based and Internet-based applications/interventions, including mobile interventions, electronic games (incl multiplayer games), social media, certain telehealth applications, and other interactive and/or networked electronic applications. Some of the items (e.g. all subitems under item 5 - description of the intervention) may also be applicable for other study designs.

The goal of the CONSORT EHEALTH checklist and guideline is to be
a) a guide for reporting for authors of RCTs,
b) to form a basis for appraisal of an ehealth trial (in terms of validity)

CONSORT-EHEALTH items/subitems are MANDATORY reporting items for studies published in the Journal of Medical Internet Research and other journals / scientific societies endorsing the checklist.

Items numbered 1., 2., 3., 4a., 4b etc are original CONSORT or CONSORT-NPT (non-pharmacologic treatment) items.
Items with Roman numerals (i., ii, iii, iv etc.) are CONSORT-EHEALTH extensions/ clarifications.

As the CONSORT-EHEALTH checklist is still considered in a formative stage, we would ask that you also RATE ON A SCALE OF 1-5 how important/useful you feel each item is FOR THE PURPOSE OF THE CHECKLIST and reporting guideline (optional).

Mandatory reporting items are marked with a red *.
In the textboxes, either copy & paste the relevant sections from your manuscript into this form - please include any quotes from your manuscript in QUOTATION MARKS,
or answer directly by providing additional information not in the manuscript, or elaborating on why the item was not relevant for this study.

YOUR ANSWERS WILL BE PUBLISHED AS A SUPPLEMENTARY FILE TO YOUR PUBLICATION IN JMIR AND ARE CONSIDERED PART OF YOUR PUBLICATION (IF ACCEPTED).
Please fill in these questions diligently. Information will not be copyedited, so please use proper spelling and grammar, use correct capitalization, and avoid abbreviations.

DO NOT FORGET TO SAVE AS PDF _AND_ CLICK THE SUBMIT BUTTON SO YOUR ANSWERS ARE IN OUR DATABASE !!!

Citation Suggestion (if you append the pdf as Appendix we suggest to cite this paper in the caption):

Eysenbach G, CONSORT-EHEALTH Group
CONSORT-EHEALTH: Improving and Standardizing Evaluation Reports of Web-based and Mobile Health Interventions
J Med Internet Res 2011;13(4):e126
URL: http://www.jmir.org/2011/4/e126/
doi: 10.2196/jmir.1923
PMID: 22209829

anakagomi0211@gmail.com
アカウントを切り替える

下書きを保存しました

共有なし

* 必須の質問です

Your name *

First Last

Atsushi Nakagomi

Primary Affiliation (short), City, Country *

University of Toronto, Toronto, Canada

Chiba University, Chiba, Japan

Your e-mail address *

abc@gmail.com

anakagomi0211@chiba-u.jp

Title of your manuscript *

Provide the (draft) title of your manuscript.

Digital Peer Support to Increase Walking Among Older Adults: A Cluster Randomized Trial

Name of your App/Software/Intervention *

If there is a short and a long/alternate name, write the short name first and add the long name in brackets.

MinChalle (A digital peer support app)

Evaluated Version (if any)

e.g. "V1", "Release 2017-03-01", "Version 2.0.27913"

回答を入力

Language(s) *

What language is the intervention/app in? If multiple languages are available, separate by comma (e.g. "English, French")

Japanese

URL of your Intervention Website or App

e.g. a direct link to the mobile app on app in appstore (itunes, Google Play), or URL of the website. If the intervention is a DVD or hardware, you can also link to an Amazon page.

回答を入力

URL of an image/screenshot (optional)

回答を入力

Accessibility *

Can an enduser access the intervention presently?

- ○ access is free and open
- ◉ access only for special usergroups, not open
- ○ access is open to everyone, but requires payment/subscription/in-app purchases
- ○ app/intervention no longer accessible
- ○ その他:

Primary Medical Indication/Disease/Condition *

e.g. "Stress", "Diabetes", or define the target group in brackets after the condition, e.g. "Autism (Parents of children with)", "Alzheimers (Informal Caregivers of)"

Community-dwelling older adults

Primary Outcomes measured in trial *

comma-separated list of primary outcomes reported in the trial

Change in weekly average of daily step counts

Secondary/other outcomes

Are there any other outcomes the intervention is expected to affect?

回答を入力

Recommended "Dose" *

What do the instructions for users say on how often the app should be used?

- (selected) Approximately Daily
- Approximately Weekly
- Approximately Monthly
- Approximately Yearly
- "as needed"
- その他:

Approx. Percentage of Users (starters) still using the app as recommended after 3 months *

- (x) unknown / not evaluated
- ( ) 0-10%
- ( ) 11-20%
- ( ) 21-30%
- ( ) 31-40%
- ( ) 41-50%
- ( ) 51-60%
- ( ) 61-70%
- ( ) 71%-80%
- ( ) 81-90%
- ( ) 91-100%
- ( ) その他:

Overall, was the app/intervention effective? *

- (●) yes: all primary outcomes were significantly better in intervention group vs control
- ( ) partly: SOME primary outcomes were significantly better in intervention group vs control
- ( ) no statistically significant difference between control and intervention
- ( ) potentially harmful: control was significantly better than intervention in one or more outcomes
- ( ) inconclusive: more research is needed
- ( ) その他:

Article Preparation Status/Stage *

At which stage in your article preparation are you currently (at the time you fill in this form)

- ( ) not submitted yet - in early draft status
- ( ) not submitted yet - in late draft status, just before submission
- ( ) submitted to a journal but not reviewed yet
- (●) submitted to a journal and after receiving initial reviewer comments
- ( ) submitted to a journal and accepted, but not published yet
- ( ) published
- ( ) その他:

Journal *

If you already know where you will submit this paper (or if it is already submitted), please provide the journal name (if it is not JMIR, provide the journal name under "other")

- ◯ not submitted yet / unclear where I will submit this
- ◉ Journal of Medical Internet Research (JMIR)
- ◯ JMIR mHealth and UHealth
- ◯ JMIR Serious Games
- ◯ JMIR Mental Health
- ◯ JMIR Public Health
- ◯ JMIR Formative Research
- ◯ Other JMIR sister journal
- ◯ その他:

Is this a full powered effectiveness trial or a pilot/feasibility trial? *

- ◯ Pilot/feasibility
- ◉ Fully powered

Manuscript tracking number *

If this is a JMIR submission, please provide the manuscript tracking number under "other" (The ms tracking number can be found in the submission acknowledgement email, or when you login as author in JMIR. If the paper is already published in JMIR, then the ms tracking number is the four-digit number at the end of the DOI, to be found at the bottom of each published article in JMIR)

- ○ no ms number (yet) / not (yet) submitted to / published in JMIR
- ◉ その他: ms#75708

TITLE AND ABSTRACT

1a) TITLE: Identification as a randomized trial in the title

1a) Does your paper address CONSORT item 1a? *

I.e does the title contain the phrase "Randomized Controlled Trial"? (if not, explain the reason under "other")

- ◉ yes
- ○ その他:

1a-i) Identify the mode of delivery in the title

Identify the mode of delivery. Preferably use “web-based” and/or “mobile” and/or “electronic game” in the title. Avoid ambiguous terms like “online”, “virtual”, “interactive”. Use “Internet-based” only if Intervention includes non-web-based Internet components (e.g. email), use “computer-based” or “electronic” only if offline products are used. Use “virtual” only in the context of “virtual reality” (3-D worlds). Use “online” only in the context of “online support groups”. Complement or substitute product names with broader terms for the class of products (such as “mobile” or “smart phone” instead of “iphone”), especially if the application runs on different platforms.

	1	2	3	4	5	
subitem not at all important	○	○	○	○	○	essential

Does your paper address subitem 1a-i? *

Copy and paste relevant sections from manuscript title (include quotes in quotation marks "like this" to indicate direct quotes from your manuscript), or elaborate on this item by providing additional information not in the ms, or briefly explain why the item is not applicable/relevant for your study

"Digital Peer Support" to Increase Physical Activity Among Older Adults: A Cluster Randomized Trial

1a-ii) Non-web-based components or important co-interventions in title

Mention non-web-based components or important co-interventions in title, if any (e.g., “with telephone support”).

	1	2	3	4	5	
subitem not at all important	○	○	○	○	○	essential

Does your paper address subitem 1a-ii?

Copy and paste relevant sections from manuscript title (include quotes in quotation marks "like this" to indicate direct quotes from your manuscript), or elaborate on this item by providing additional information not in the ms, or briefly explain why the item is not applicable/relevant for your study

回答を入力

1a-iii) Primary condition or target group in the title

Mention primary condition or target group in the title, if any (e.g., “for children with Type I Diabetes”) Example: A Web-based and Mobile Intervention with Telephone Support for Children with Type I Diabetes: Randomized Controlled Trial

	1	2	3	4	5	
subitem not at all important	○	○	○	○	○	essential

Does your paper address subitem 1a-iii? *

Copy and paste relevant sections from manuscript title (include quotes in quotation marks "like this" to indicate direct quotes from your manuscript), or elaborate on this item by providing additional information not in the ms, or briefly explain why the item is not applicable/relevant for your study

Digital Peer Support to Increase Physical Activity Among "Older Adults": A Cluster Randomized Trial

1b) ABSTRACT: Structured summary of trial design, methods, results, and conclusions

NPT extension: Description of experimental treatment, comparator, care providers, centers, and blinding status.

1b-i) Key features/functionalities/components of the intervention and comparator in the METHODS section of the ABSTRACT

Mention key features/functionalities/components of the intervention and comparator in the abstract. If possible, also mention theories and principles used for designing the site. Keep in mind the needs of systematic reviewers and indexers by including important synonyms. (Note: Only report in the abstract what the main paper is reporting. If this information is missing from the main body of text, consider adding it)

	1	2	3	4	5	
subitem not at all important	○	○	○	○	○	essential

Does your paper address subitem 1b-i? *

Copy and paste relevant sections from the manuscript abstract (include quotes in quotation marks "like this" to indicate direct quotes from your manuscript), or elaborate on this item by providing additional information not in the ms, or briefly explain why the item is not applicable/relevant for your study

All participants received a baseline smartphone lecture. Intervention participants attended two additional sessions using a digital peer support app (MinChalle), which included features such as daily step goals, peer sharing, and group encouragement. Control participants attended two standard follow-up smartphone lectures.

1b-ii) Level of human involvement in the METHODS section of the ABSTRACT

Clarify the level of human involvement in the abstract, e.g., use phrases like “fully automated” vs. “therapist/nurse/care provider/physician-assisted” (mention number and expertise of providers involved, if any). (Note: Only report in the abstract what the main paper is reporting. If this information is missing from the main body of text, consider adding it)

	1	2	3	4	5	
subitem not at all important	○	○	○	○	○	essential

Does your paper address subitem 1b-ii?

Copy and paste relevant sections from the manuscript abstract (include quotes in quotation marks "like this" to indicate direct quotes from your manuscript), or elaborate on this item by providing additional information not in the ms, or briefly explain why the item is not applicable/relevant for your study

回答を入力

1b-iii) Open vs. closed, web-based (self-assessment) vs. face-to-face assessments in the METHODS section of the ABSTRACT

Mention how participants were recruited (online vs. offline), e.g., from an open access website or from a clinic or a closed online user group (closed usergroup trial), and clarify if this was a purely web-based trial, or there were face-to-face components (as part of the intervention or for assessment). Clearly say if outcomes were self-assessed through questionnaires (as common in web-based trials). Note: In traditional offline trials, an open trial (open-label trial) is a type of clinical trial in which both the researchers and participants know which treatment is being administered. To avoid confusion, use “blinded” or “unblinded” to indicated the level of blinding instead of “open”, as “open” in web-based trials usually refers to “open access” (i.e. participants can self-enrol). (Note: Only report in the abstract what the main paper is reporting. If this information is missing from the main body of text, consider adding it)

	1	2	3	4	5	
subitem not at all important	○	○	○	○	○	essential

Does your paper address subitem 1b-iii?

Copy and paste relevant sections from the manuscript abstract (include quotes in quotation marks "like this" to indicate direct quotes from your manuscript), or elaborate on this item by providing additional information not in the ms, or briefly explain why the item is not applicable/relevant for your study

Participants were recruited offline during community smartphone lectures (closed-group recruitment). The intervention combined face-to-face lectures with app-based peer support, while outcomes were assessed both objectively (via smartphones) and through self-administered paper questionnaires.

1b-iv) RESULTS section in abstract must contain use data

Report number of participants enrolled/assessed in each group, the use/uptake of the intervention (e.g., attrition/adherence metrics, use over time, number of logins etc.), in addition to primary/secondary outcomes. (Note: Only report in the abstract what the main paper is reporting. If this information is missing from the main body of text, consider adding it)

	1	2	3	4	5	
subitem not at all important	○	○	○	○	○	essential

Does your paper address subitem 1b-iv?

Copy and paste relevant sections from the manuscript abstract (include quotes in quotation marks "like this" to indicate direct quotes from your manuscript), or elaborate on this item by providing additional information not in the ms, or briefly explain why the item is not applicable/relevant for your study

A total of 156 community-dwelling older adults were grouped into 40 clusters and randomized (20 intervention clusters [n = 80], 20 control clusters [n = 76]). In total, 124 participants (79.5%) completed the follow-up, and valid step data were available for 117 participants, with missing data ranging from 5.1% to 29.1%.

1b-v) CONCLUSIONS/DISCUSSION in abstract for negative trials

Conclusions/Discussions in abstract for negative trials: Discuss the primary outcome - if the trial is negative (primary outcome not changed), and the intervention was not used, discuss whether negative results are attributable to lack of uptake and discuss reasons. (Note: Only report in the abstract what the main paper is reporting. If this information is missing from the main body of text, consider adding it)

	1	2	3	4	5	
subitem not at all important	○	○	○	○	○	essential

Does your paper address subitem 1b-v?

Copy and paste relevant sections from the manuscript abstract (include quotes in quotation marks "like this" to indicate direct quotes from your manuscript), or elaborate on this item by providing additional information not in the ms, or briefly explain why the item is not applicable/relevant for your study

回答を入力

INTRODUCTION

2a) In INTRODUCTION: Scientific background and explanation of rationale

2a-i) Problem and the type of system/solution

Describe the problem and the type of system/solution that is object of the study: intended as stand-alone intervention vs. incorporated in broader health care program? Intended for a particular patient population? Goals of the intervention, e.g., being more cost-effective to other interventions, replace or complement other solutions? (Note: Details about the intervention are provided in “Methods” under 5)

	1	2	3	4	5	
subitem not at all important	○	○	○	○	○	essential

Does your paper address subitem 2a-i? *

Copy and paste relevant sections from the manuscript (include quotes in quotation marks "like this" to indicate direct quotes from your manuscript), or elaborate on this item by providing additional information not in the ms, or briefly explain why the item is not applicable/relevant for your study

The manuscript describes the problem of physical inactivity among older adults in Japan, where a rapidly aging population raises concerns about healthcare costs, and identifies scalable strategies to promote physical activity as an urgent need (Introduction, para 1). The solution tested is a smartphone-based digital peer support intervention (MinChalle), delivered alongside conventional smartphone lectures. It is intended as a stand-alone digital health program for community-dwelling older adults (≥60 years), with the goal of promoting physical activity through behavior change techniques and peer support mechanisms (Introduction, paras 2–4).

2a-ii) Scientific background, rationale: What is known about the (type of) system

Scientific background, rationale: What is known about the (type of) system that is the object of the study (be sure to discuss the use of similar systems for other conditions/ diagnoses, if appropiate), motivation for the study, i.e. what are the reasons for and what is the context for this specific study, from which stakeholder viewpoint is the study performed, potential impact of findings [2]. Briefly justify the choice of the comparator.

	1	2	3	4	5	
subitem not at all important	○	○	○	○	○	essential

Does your paper address subitem 2a-ii? *

Copy and paste relevant sections from the manuscript (include quotes in quotation marks "like this" to indicate direct quotes from your manuscript), or elaborate on this item by providing additional information not in the ms, or briefly explain why the item is not applicable/relevant for your study

With the growth of digital health technologies, many mobile applications now integrate behavior change techniques[14]. Alongside intrapersonal features, peer-support elements such as group challenges and shared goals are increasingly common[15-17]. For example, peer support can be structured in a smartphone app (Minchalle) through small groups of three to five participants who collaboratively select step goals, commit to posting updates at regular intervals, and encourage each other's progress [17, 18]. Integrating such peer support with intrapersonal strategies within the app has been shown to enhance user engagement and adherence to health goals [17, 18].

However, older adults often face barriers—known as the "digital divide"—that hinder their use of technology-based interventions[19]. In Japan, local governments and community groups have introduced smartphone lectures to bridge this gap, yet sustained smartphone use remains a challenge. Digital peer support may offer a more lasting solution by providing ongoing group-based reinforcement, promoting both technology use and healthier behaviors. Still, few studies have compared smartphone lectures with and without digital peer support, highlighting a gap in empirical evidence.

This study evaluates whether adding digital peer support to smartphone lectures increases physical activity among older Japanese adults. Using a cluster-randomized controlled trial, we compared a conventional smartphone lecture to one combined with a peer support app grounded in behavioral science. A cluster design was required because the intervention was implemented within peer groups of three to five participants; randomizing individuals within the same group would have compromised the integrity of the peer support mechanism and risked contamination between trial arms. The primary objective of the trial was to evaluate changes in daily step counts at the individual participant level, while randomization occurred at the cluster (group) level to preserve the integrity of the peer support intervention. Participants were recruited in community settings and randomly assigned to either group. This design allowed us to examine whether ongoing digital peer support enhances motivation, sustains smartphone engagement, and improves physical activity outcomes.

2b) In INTRODUCTION: Specific objectives or hypotheses

Does your paper address CONSORT subitem 2b? *

Copy and paste relevant sections from the manuscript (include quotes in quotation marks "like this" to indicate direct quotes from your manuscript), or elaborate on this item by providing additional information not in the ms, or briefly explain why the item is not applicable/relevant for your study

The primary objective of the trial was to evaluate changes in daily step counts at the individual participant level, while randomization occurred at the cluster (group) level to preserve the integrity of the peer support intervention.

METHODS

3a) Description of trial design (such as parallel, factorial) including allocation ratio

Does your paper address CONSORT subitem 3a? *

Copy and paste relevant sections from the manuscript (include quotes in quotation marks "like this" to indicate direct quotes from your manuscript), or elaborate on this item by providing additional information not in the ms, or briefly explain why the item is not applicable/relevant for your study

This was a 2-arm, 1:1 parallel-group, cluster-randomized trial that included a 2-week baseline measurement period followed by a 12-week intervention period. The unit of randomization (the cluster) was a peer support group consisting of three to five participants. In the intervention arm, these clusters functioned as peer groups within the MinChalle app, while in the control arm participants were grouped to maintain comparability but did not engage in peer support during the trial. Control participants were informed that they would be offered the opportunity to form peer support groups after the trial (waiting-list design). Randomization at the cluster level was required to preserve the integrity of group allocation.

3b) Important changes to methods after trial commencement (such as eligibility criteria), with reasons

Does your paper address CONSORT subitem 3b? *

Copy and paste relevant sections from the manuscript (include quotes in quotation marks "like this" to indicate direct quotes from your manuscript), or elaborate on this item by providing additional information not in the ms, or briefly explain why the item is not applicable/relevant for your study

No significant methodological changes were made.

3b-i) Bug fixes, Downtimes, Content Changes

Bug fixes, Downtimes, Content Changes: ehealth systems are often dynamic systems. A description of changes to methods therefore also includes important changes made on the intervention or comparator during the trial (e.g., major bug fixes or changes in the functionality or content) (5-iii) and other “unexpected events” that may have influenced study design such as staff changes, system failures/downtimes, etc. [2].

	1	2	3	4	5	
subitem not at all important	○	○	○	○	○	essential

Does your paper address subitem 3b-i?

Copy and paste relevant sections from the manuscript (include quotes in quotation marks "like this" to indicate direct quotes from your manuscript), or elaborate on this item by providing additional information not in the ms, or briefly explain why the item is not applicable/relevant for your study

No important changes were made on the intervention during the trial.

4a) Eligibility criteria for participants

Does your paper address CONSORT subitem 4a? *

Copy and paste relevant sections from the manuscript (include quotes in quotation marks "like this" to indicate direct quotes from your manuscript), or elaborate on this item by providing additional information not in the ms, or briefly explain why the item is not applicable/relevant for your study

"Participants were eligible if they (1) were ≥60 years old, (2) could communicate in Japanese, (3) could read and provide informed consent, and (4) owned a smartphone at the time of consent. Participants were excluded if they (1) had any condition making participation infeasible (e.g., inability to walk with a smartphone), (2) had a medical restriction on walking (e.g., per doctor's advice), or (3) had already used the Minchalle app. All participants provided written informed consent before enrolment."

4a-i) Computer / Internet literacy

Computer / Internet literacy is often an implicit “de facto” eligibility criterion - this should be explicitly clarified.

	1	2	3	4	5	
subitem not at all important	○	○	○	○	○	essential

Does your paper address subitem 4a-i?

Copy and paste relevant sections from the manuscript (include quotes in quotation marks "like this" to indicate direct quotes from your manuscript), or elaborate on this item by providing additional information not in the ms, or briefly explain why the item is not applicable/relevant for your study

回答を入力

4a-ii) Open vs. closed, web-based vs. face-to-face assessments:

Open vs. closed, web-based vs. face-to-face assessments: Mention how participants were recruited (online vs. offline), e.g., from an open access website or from a clinic, and clarify if this was a purely web-based trial, or there were face-to-face components (as part of the intervention or for assessment), i.e., to what degree got the study team to know the participant. In online-only trials, clarify if participants were quasi-anonymous and whether having multiple identities was possible or whether technical or logistical measures (e.g., cookies, email confirmation, phone calls) were used to detect/prevent these.

	1	2	3	4	5	
subitem not at all important	○	○	○	○	○	essential

Does your paper address subitem 4a-ii? *

Copy and paste relevant sections from the manuscript (include quotes in quotation marks "like this" to indicate direct quotes from your manuscript), or elaborate on this item by providing additional information not in the ms, or briefly explain why the item is not applicable/relevant for your study

“Recruitment took place from September 2023 to June 2024, in partnership with the Sumida Ward local government and senior clubs in Chiba City. Individuals were recruited in community venues during smartphone lectures hosted by the local municipal government or senior clubs, which aimed to bridge the digital divide and promote physical activity to prevent frailty among older adults.”

“All participants provided written informed consent before enrolment.”

4a-iii) Information giving during recruitment

Information given during recruitment. Specify how participants were briefed for recruitment and in the informed consent procedures (e.g., publish the informed consent documentation as appendix, see also item X26), as this information may have an effect on user self-selection, user expectation and may also bias results.

	1	2	3	4	5	
subitem not at all important	○	○	○	○	○	essential

Does your paper address subitem 4a-iii?

Copy and paste relevant sections from the manuscript (include quotes in quotation marks "like this" to indicate direct quotes from your manuscript), or elaborate on this item by providing additional information not in the ms, or briefly explain why the item is not applicable/relevant for your study

回答を入力

4b) Settings and locations where the data were collected

Does your paper address CONSORT subitem 4b? *

Copy and paste relevant sections from the manuscript (include quotes in quotation marks "like this" to indicate direct quotes from your manuscript), or elaborate on this item by providing additional information not in the ms, or briefly explain why the item is not applicable/relevant for your study

“Recruitment took place from September 2023 to June 2024, in partnership with the Sumida Ward local government and senior clubs in Chiba City. Individuals were recruited in community venues during smartphone lectures hosted by the local municipal government or senior clubs, which aimed to bridge the digital divide and promote physical activity to prevent frailty among older adults. The local government and senior clubs outsourced lecture services to A10 Lab Inc, developer of the ‘Minchalle’ app (Challenge with All), and Active Senior Outreach Nonprofit Organization, a nonprofit organization specializing in smartphone lectures for older adults.”

4b-i) Report if outcomes were (self-)assessed through online questionnaires

Clearly report if outcomes were (self-)assessed through online questionnaires (as common in web-based trials) or otherwise.

	1	2	3	4	5	
subitem not at all important	○	○	○	○	○	essential

Does your paper address subitem 4b-i? *

Copy and paste relevant sections from the manuscript (include quotes in quotation marks "like this" to indicate direct quotes from your manuscript), or elaborate on this item by providing additional information not in the ms, or briefly explain why the item is not applicable/relevant for your study

“After the baseline assessment, participants were asked to carry their smartphones at all times to capture daily steps.”

“The IPAQ short form was administered at baseline and follow-up to assess frequency and duration of vigorous, moderate, and walking activities (≥10-minute bouts).”

4b-ii) Report how institutional affiliations are displayed

Report how institutional affiliations are displayed to potential participants [on ehealth media], as affiliations with prestigious hospitals or universities may affect volunteer rates, use, and reactions with regards to an intervention.(Not a required item – describe only if this may bias results)

	1	2	3	4	5	
subitem not at all important	○	○	○	○	○	essential

Does your paper address subitem 4b-ii?

Copy and paste relevant sections from the manuscript (include quotes in quotation marks "like this" to indicate direct quotes from your manuscript), or elaborate on this item by providing additional information not in the ms, or briefly explain why the item is not applicable/relevant for your study

回答を入力

5) The interventions for each group with sufficient details to allow replication, including how and when they were actually administered

5-i) Mention names, credential, affiliations of the developers, sponsors, and owners

Mention names, credential, affiliations of the developers, sponsors, and owners [6] (if authors/evaluators are owners or developer of the software, this needs to be declared in a “Conflict of interest” section or mentioned elsewhere in the manuscript).

	1	2	3	4	5	
subitem not at all important	○	○	○	○	○	essential

Does your paper address subitem 5-i?

Copy and paste relevant sections from the manuscript (include quotes in quotation marks "like this" to indicate direct quotes from your manuscript), or elaborate on this item by providing additional information not in the ms, or briefly explain why the item is not applicable/relevant for your study

“The app, developed by A10 Lab Inc. and launched in 2015, was provided free of charge.”

“This study was funded by A10 Lab Inc.”

“This study was funded by A10 Lab Inc. Atsushi Nakagomi and Katsunori Kondo have received research funding from A10 Lab Inc. to evaluate the MinChalle app and provide feedback on service improvement based on study findings. The sponsor’s role was limited to introducing municipalities for recruitment and providing raw step count data. The sponsor had no involvement in the study design, data analysis, data interpretation, manuscript writing, or the decision to submit the article for publication. To safeguard independence, all statistical analyses were conducted solely by academic investigators (NA and AN), with independent oversight by YK.”

5-ii) Describe the history/development process

Describe the history/development process of the application and previous formative evaluations (e.g., focus groups, usability testing), as these will have an impact on adoption/use rates and help with interpreting results.

	1	2	3	4	5	
subitem not at all important	○	○	○	○	○	essential

Does your paper address subitem 5-ii?

Copy and paste relevant sections from the manuscript (include quotes in quotation marks "like this" to indicate direct quotes from your manuscript), or elaborate on this item by providing additional information not in the ms, or briefly explain why the item is not applicable/relevant for your study

回答を入力

5-iii) Revisions and updating

Revisions and updating. Clearly mention the date and/or version number of the application/intervention (and comparator, if applicable) evaluated, or describe whether the intervention underwent major changes during the evaluation process, or whether the development and/or content was "frozen" during the trial. Describe dynamic components such as news feeds or changing content which may have an impact on the replicability of the intervention (for unexpected events see item 3b).

	1	2	3	4	5	
subitem not at all important	○	○	○	○	○	essential

Does your paper address subitem 5-iii?

Copy and paste relevant sections from the manuscript (include quotes in quotation marks "like this" to indicate direct quotes from your manuscript), or elaborate on this item by providing additional information not in the ms, or briefly explain why the item is not applicable/relevant for your study

回答を入力

5-iv) Quality assurance methods

Provide information on quality assurance methods to ensure accuracy and quality of information provided [1], if applicable.

	1	2	3	4	5	
subitem not at all important	○	○	○	○	○	essential

Does your paper address subitem 5-iv?

Copy and paste relevant sections from the manuscript (include quotes in quotation marks "like this" to indicate direct quotes from your manuscript), or elaborate on this item by providing additional information not in the ms, or briefly explain why the item is not applicable/relevant for your study

回答を入力

5-v) Ensure replicability by publishing the source code, and/or providing screenshots/screen-capture video, and/or providing flowcharts of the algorithms used

Ensure replicability by publishing the source code, and/or providing screenshots/screen-capture video, and/or providing flowcharts of the algorithms used. Replicability (i.e., other researchers should in principle be able to replicate the study) is a hallmark of scientific reporting.

	1	2	3	4	5	
subitem not at all important	○	○	○	○	○	essential

Does your paper address subitem 5-v?

Copy and paste relevant sections from the manuscript (include quotes in quotation marks "like this" to indicate direct quotes from your manuscript), or elaborate on this item by providing additional information not in the ms, or briefly explain why the item is not applicable/relevant for your study

回答を入力

5-vi) Digital preservation

Digital preservation: Provide the URL of the application, but as the intervention is likely to change or disappear over the course of the years; also make sure the intervention is archived (Internet Archive, webcitation.org, and/or publishing the source code or screenshots/videos alongside the article). As pages behind login screens cannot be archived, consider creating demo pages which are accessible without login.

	1	2	3	4	5	
subitem not at all important	○	○	○	○	○	essential

Does your paper address subitem 5-vi?

Copy and paste relevant sections from the manuscript (include quotes in quotation marks "like this" to indicate direct quotes from your manuscript), or elaborate on this item by providing additional information not in the ms, or briefly explain why the item is not applicable/relevant for your study

回答を入力

5-vii) Access

Access: Describe how participants accessed the application, in what setting/context, if they had to pay (or were paid) or not, whether they had to be a member of specific group. If known, describe how participants obtained "access to the platform and Internet" [1]. To ensure access for editors/reviewers/readers, consider to provide a "backdoor" login account or demo mode for reviewers/readers to explore the application (also important for archiving purposes, see vi).

	1	2	3	4	5	
subitem not at all important	○	○	○	○	○	essential

Does your paper address subitem 5-vii? *

Copy and paste relevant sections from the manuscript (include quotes in quotation marks "like this" to indicate direct quotes from your manuscript), or elaborate on this item by providing additional information not in the ms, or briefly explain why the item is not applicable/relevant for your study

"The intervention was a smartphone lecture program incorporating the digital peer support app MinChalle. All smartphone lecture programs were conducted in community venues in Sumida Ward (Tokyo) and Chiba City (Chiba Prefecture), which are typical venues for older adult smartphone lectures. The app, developed by A10 Lab Inc. and launched in 2015, was provided free of charge."

"Two weeks after the initial baseline session, participants in the intervention arm attended two additional weekly lectures led by A10 Lab Inc., the developer of MinChalle. In these lectures, participants were instructed on how to install the app and configure initial settings, including selecting group step goals and defining posting intervals … They also learned how to take photos, type messages, and post them in the group chat, supported by manuals provided during the sessions."

"Participants were eligible if they … owned a smartphone at the time of consent."

5-viii) Mode of delivery, features/functionalities/components of the intervention and comparator, and the theoretical framework

Describe mode of delivery, features/functionalities/components of the intervention and comparator, and the theoretical framework [6] used to design them (instructional strategy [1], behaviour change techniques, persuasive features, etc., see e.g., [7, 8] for terminology). This includes an in-depth description of the content (including where it is coming from and who developed it) [1],” whether [and how] it is tailored to individual circumstances and allows users to track their progress and receive feedback” [6]. This also includes a description of communication delivery channels and – if computer-mediated communication is a component – whether communication was synchronous or asynchronous [6]. It also includes information on presentation strategies [1], including page design principles, average amount of text on pages, presence of hyperlinks to other resources, etc. [1].

	1	2	3	4	5	
subitem not at all important	○	○	○	○	○	essential

Does your paper address subitem 5-viii? *

Copy and paste relevant sections from the manuscript (include quotes in quotation marks "like this" to indicate direct quotes from your manuscript), or elaborate on this item by providing additional information not in the ms, or briefly explain why the item is not applicable/relevant for your study

The manuscript states:

“The intervention was a smartphone lecture program incorporating the digital peer support app MinChalle. All smartphone lecture programs were conducted in community venues in Sumida Ward (Tokyo) and Chiba City (Chiba Prefecture), which are typical venues for older adult smartphone lectures. The app, developed by A10 Lab Inc. and launched in 2015, was provided free of charge. Illustrations of the app are shown in Supplementary Figure 1. A structured description of the intervention is also provided according to the TIDieR checklist (Supplementary Table 2).
MinChalle integrates several behavior change techniques (BCTs) as defined by the BCT Taxonomy v1 [22]. The core element of MinChalle was the peer support component, in which groups of three to five members supported one another to achieve their goals by posting at least one photo and one message daily in the group chat (social support: general [BCT 3.1]). In addition, groups of three to five members selected daily step goals of 2,500, 5,000, or 10,000 steps (goal setting [BCT 1.1] and action planning [BCT 1.4]). Groups also selected a posting interval (every 4, 8, or 15 days). Members who failed to post within the agreed interval were removed (with the option to rejoin), reinforcing commitment [BCT 1.9: commitment]. Step counts were automatically tracked and displayed in the chat, enabling self-monitoring of behavior [BCT 2.3] and promoting feedback and social comparison [BCT 6.2, 6.3] through the visibility of group achievements. This design was grounded in Social Cognitive Theory (SCT), where peer encouragement, observational learning, and reinforcement are expected to enhance self-efficacy and sustain physical activity.

Two weeks after the initial baseline session, participants in the intervention arm attended two additional weekly lectures led by A10 Lab Inc., the developer of MinChalle. In these lectures, participants were instructed on how to install the app and configure initial settings, including selecting group step goals and defining posting intervals, after which members who failed to post within the interval would be removed.. They also learned how to take photos, type messages, and post them in the group chat, supported by manuals provided during the sessions. ”

For the control group, the manuscript states:

“Participants in the control arm also attended two smartphone lectures, akin to typical sessions for older adults in Japan. These lectures covered basic smartphone functions—including email, web browsing, use of the messenger app LINE, and official local government apps or accounts. As in the initial lecture, they were encouraged to continue using Google Fit or Apple Health for step tracking.”

5-ix) Describe use parameters

Describe use parameters (e.g., intended “doses” and optimal timing for use). Clarify what instructions or recommendations were given to the user, e.g., regarding timing, frequency, heaviness of use, if any, or was the intervention used ad libitum.

	1	2	3	4	5	
subitem not at all important	○	○	○	○	○	essential

Does your paper address subitem 5-ix?

Copy and paste relevant sections from the manuscript (include quotes in quotation marks "like this" to indicate direct quotes from your manuscript), or elaborate on this item by providing additional information not in the ms, or briefly explain why the item is not applicable/relevant for your study

回答を入力

5-x) Clarify the level of human involvement

Clarify the level of human involvement (care providers or health professionals, also technical assistance) in the e-intervention or as co-intervention (detail number and expertise of professionals involved, if any, as well as “type of assistance offered, the timing and frequency of the support, how it is initiated, and the medium by which the assistance is delivered”. It may be necessary to distinguish between the level of human involvement required for the trial, and the level of human involvement required for a routine application outside of a RCT setting (discuss under item 21 – generalizability).

	1	2	3	4	5	
subitem not at all important	○	○	○	○	○	essential

Does your paper address subitem 5-x?

Copy and paste relevant sections from the manuscript (include quotes in quotation marks "like this" to indicate direct quotes from your manuscript), or elaborate on this item by providing additional information not in the ms, or briefly explain why the item is not applicable/relevant for your study

回答を入力

5-xi) Report any prompts/reminders used

Report any prompts/reminders used: Clarify if there were prompts (letters, emails, phone calls, SMS) to use the application, what triggered them, frequency etc. It may be necessary to distinguish between the level of prompts/reminders required for the trial, and the level of prompts/reminders for a routine application outside of a RCT setting (discuss under item 21 – generalizability).

	1	2	3	4	5	
subitem not at all important	○	○	○	○	○	essential

Does your paper address subitem 5-xi? *

Copy and paste relevant sections from the manuscript (include quotes in quotation marks "like this" to indicate direct quotes from your manuscript), or elaborate on this item by providing additional information not in the ms, or briefly explain why the item is not applicable/relevant for your study

“Groups also selected a posting interval (every 4, 8, or 15 days). Members who failed to post within the agreed interval were removed (with the option to rejoin), reinforcing commitment [BCT 1.9: commitment]. ”

No additional prompts or reminders (e.g., emails, phone calls, or SMS) were used by the research team during the trial. All reminder mechanisms were built into the MinChalle app itself, through the posting interval rules.

5-xii) Describe any co-interventions (incl. training/support)

Describe any co-interventions (incl. training/support): Clearly state any interventions that are provided in addition to the targeted eHealth intervention, as ehealth intervention may not be designed as stand-alone intervention. This includes training sessions and support [1]. It may be necessary to distinguish between the level of training required for the trial, and the level of training for a routine application outside of a RCT setting (discuss under item 21 – generalizability.

	1	2	3	4	5	
subitem not at all important	○	○	○	○	○	essential

Does your paper address subitem 5-xii? *

Copy and paste relevant sections from the manuscript (include quotes in quotation marks "like this" to indicate direct quotes from your manuscript), or elaborate on this item by providing additional information not in the ms, or briefly explain why the item is not applicable/relevant for your study

“Two weeks after the initial baseline session, participants in the intervention arm attended two additional weekly lectures led by A10 Lab Inc., the developer of MinChalle. In these lectures, participants were instructed on how to install the app and configure initial settings, including selecting group step goals and defining posting intervals … They also learned how to take photos, type messages, and post them in the group chat, supported by manuals provided during the sessions.”

6a) Completely defined pre-specified primary and secondary outcome measures, including how and when they were assessed

Does your paper address CONSORT subitem 6a? *

Copy and paste relevant sections from the manuscript (include quotes in quotation marks "like this" to indicate direct quotes from your manuscript), or elaborate on this item by providing additional information not in the ms, or briefly explain why the item is not applicable/relevant for your study

"The primary outcome was the change in the weekly average of daily step counts from baseline to week 12 (three months after installation of the peer support app) . After the baseline assessment, participants were asked to carry their smartphones at all times to capture daily steps. During the 2-week baseline period leading up to the second lecture, baseline step counts were measured. To minimize mismeasurement, any day with fewer than 1000 steps was treated as missing [23]. No changes to pre-specified primary outcome were made after trial commencement. All primary outcome data pertained to the individual participant level, although randomization was conducted at the cluster level.

Secondary outcomes included weekly step counts at other intervention weeks (weeks 1–11) and monthly averages (months 1–3), total physical activity (Metabolic Equivalent of Task (MET) assessed by the International Physical Activity Questionnaire [IPAQ] short form)) [24], self-reported walking time (≥30 minutes/day), daily smartphone use, and the number of smartphone use purposes at follow-up. The IPAQ short form was administered at baseline and follow-up to assess frequency and duration of vigorous, moderate, and walking activities (≥10-minute bouts). Walking time was dichotomized as <30 or ≥30 minutes/day. Smartphone use was dichotomized as daily (almost daily) vs. not daily. Smartphone use purposes were assessed from a checklist and summed to yield a total score from information search and browsing, work-related tasks, communication (e.g., email, LINE, video calls), taking photos or videos, using maps or traffic information, online banking, shopping, social media (e.g., Facebook, Twitter), health apps (e.g., step counters), entertainment (e.g., video streaming, e-books, music), gaming, and other uses."

6a-i) Online questionnaires: describe if they were validated for online use and apply CHERRIES items to describe how the questionnaires were designed/deployed

If outcomes were obtained through online questionnaires, describe if they were validated for online use and apply CHERRIES items to describe how the questionnaires were designed/deployed [9].

	1	2	3	4	5	
subitem not at all important	○	○	○	○	○	essential

Does your paper address subitem 6a-i?

Copy and paste relevant sections from manuscript text

回答を入力

6a-ii) Describe whether and how “use” (including intensity of use/dosage) was defined/measured/monitored

Describe whether and how “use” (including intensity of use/dosage) was defined/measured/monitored (logins, logfile analysis, etc.). Use/adoption metrics are important process outcomes that should be reported in any ehealth trial.

	1	2	3	4	5	
subitem not at all important	○	○	○	○	○	essential

Does your paper address subitem 6a-ii?

Copy and paste relevant sections from manuscript text

回答を入力

6a-iii) Describe whether, how, and when qualitative feedback from participants was obtained

Describe whether, how, and when qualitative feedback from participants was obtained (e.g., through emails, feedback forms, interviews, focus groups).

	1	2	3	4	5	
subitem not at all important	○	○	○	○	○	essential

Does your paper address subitem 6a-iii?

Copy and paste relevant sections from manuscript text

回答を入力

6b) Any changes to trial outcomes after the trial commenced, with reasons

Does your paper address CONSORT subitem 6b? *

Copy and paste relevant sections from the manuscript (include quotes in quotation marks "like this" to indicate direct quotes from your manuscript), or elaborate on this item by providing additional information not in the ms, or briefly explain why the item is not applicable/relevant for your study

No changes to pre-specified primary outcome were made after trial commencement.

7a) How sample size was determined

NPT: When applicable, details of whether and how the clustering by care provides or centers was addressed

7a-i) Describe whether and how expected attrition was taken into account when calculating the sample size

Describe whether and how expected attrition was taken into account when calculating the sample size.

	1	2	3	4	5	
subitem not at all important	○	○	○	○	○	essential

Does your paper address subitem 7a-i?

Copy and paste relevant sections from manuscript title (include quotes in quotation marks "like this" to indicate direct quotes from your manuscript), or elaborate on this item by providing additional information not in the ms, or briefly explain why the item is not applicable/relevant for your study

回答を入力

7b) When applicable, explanation of any interim analyses and stopping guidelines

Does your paper address CONSORT subitem 7b? *

Copy and paste relevant sections from the manuscript (include quotes in quotation marks "like this" to indicate direct quotes from your manuscript), or elaborate on this item by providing additional information not in the ms, or briefly explain why the item is not applicable/relevant for your study

“An interim analysis was pre-specified to be conducted after the enrollment of approximately half of the planned sample size (n ≈ 100). The trial was to be stopped if statistically significant differences in the primary outcome (change in step counts between baseline and week 12) were observed. ”

8a) Method used to generate the random allocation sequence

NPT: When applicable, how care providers were allocated to each trial group

Does your paper address CONSORT subitem 8a? *

Copy and paste relevant sections from the manuscript (include quotes in quotation marks "like this" to indicate direct quotes from your manuscript), or elaborate on this item by providing additional information not in the ms, or briefly explain why the item is not applicable/relevant for your study

“Following the initial session and baseline assessment, randomization was conducted at the cluster level, with each group of three to five participants serving as the unit of allocation. The random allocation sequence was generated electronically using a random number generator with block sizes of 2 to 4 clusters.”

8b) Type of randomisation; details of any restriction (such as blocking and block size)

Does your paper address CONSORT subitem 8b? *

Copy and paste relevant sections from the manuscript (include quotes in quotation marks "like this" to indicate direct quotes from your manuscript), or elaborate on this item by providing additional information not in the ms, or briefly explain why the item is not applicable/relevant for your study

“Following the initial session and baseline assessment, randomization was conducted at the cluster level, with each group of three to five participants serving as the unit of allocation. The random allocation sequence was generated electronically using a random number generator with block sizes of 2 to 4 clusters.”

9) Mechanism used to implement the random allocation sequence (such as sequentially numbered containers), describing any steps taken to conceal the sequence until interventions were assigned

Does your paper address CONSORT subitem 9? *

Copy and paste relevant sections from the manuscript (include quotes in quotation marks "like this" to indicate direct quotes from your manuscript), or elaborate on this item by providing additional information not in the ms, or briefly explain why the item is not applicable/relevant for your study

“The random allocation sequence was generated electronically using a random number generator with block sizes of 2 to 4 clusters. An independent allocation officer from the Institute for Assistance of Academic and Education implemented the sequence. The allocation sequence and block size were concealed from study personnel during enrolment, and group assignments were revealed only after enrolment was finalized.”

10) Who generated the random allocation sequence, who enrolled participants, and who assigned participants to interventions

Does your paper address CONSORT subitem 10? *

Copy and paste relevant sections from the manuscript (include quotes in quotation marks "like this" to indicate direct quotes from your manuscript), or elaborate on this item by providing additional information not in the ms, or briefly explain why the item is not applicable/relevant for your study

“The random allocation sequence was generated electronically using a random number generator with block sizes of 2 to 4 clusters. An independent allocation officer from the Institute for Assistance of Academic and Education implemented the sequence. The allocation sequence and block size were concealed from study personnel during enrolment, and group assignments were revealed only after enrolment was finalized.”

“Individual participants were enrolled during community smartphone lectures and grouped pragmatically into clusters of three to five based on attendance at the same session. Informed consent was obtained directly from all individual participants prior to randomization. Randomization was therefore performed at the cluster level, while allocation concealment and consent were handled at the individual participant level.”

11a) If done, who was blinded after assignment to interventions (for example, participants, care providers, those assessing outcomes) and how

NPT: Whether or not administering co-interventions were blinded to group assignment

11a-i) Specify who was blinded, and who wasn't

Specify who was blinded, and who wasn't. Usually, in web-based trials it is not possible to blind the participants [1, 3] (this should be clearly acknowledged), but it may be possible to blind outcome assessors, those doing data analysis or those administering co-interventions (if any).

	1	2	3	4	5	
subitem not at all important	○	○	○	○	○	essential

Does your paper address subitem 11a-i? *

Copy and paste relevant sections from the manuscript (include quotes in quotation marks "like this" to indicate direct quotes from your manuscript), or elaborate on this item by providing additional information not in the ms, or briefly explain why the item is not applicable/relevant for your study

“Because of the nature of the intervention, participants and local staff could not be blinded to group assignment. However, outcome assessment was based on step count data automatically recorded by participants' smartphones, and data analysts were blinded to allocation, reducing the risk of bias.
The two study arms differed in that only the intervention group received access to the MinChalle app and related training. Therefore, the interventions could not be made similar in appearance or content.”

11a-ii) Discuss e.g., whether participants knew which intervention was the "intervention of interest" and which one was the "comparator"

Informed consent procedures (4a-ii) can create biases and certain expectations - discuss e.g., whether participants knew which intervention was the "intervention of interest" and which one was the "comparator".

	1	2	3	4	5	
subitem not at all important	○	○	○	○	○	essential

Does your paper address subitem 11a-ii?

Copy and paste relevant sections from the manuscript (include quotes in quotation marks "like this" to indicate direct quotes from your manuscript), or elaborate on this item by providing additional information not in the ms, or briefly explain why the item is not applicable/relevant for your study

"Because of the nature of the intervention, participants could not be blinded to group assignment. However, outcome assessment was based on step count data automatically recorded by participants' smartphones, which reduced the potential for assessor bias. Analyses were performed by investigators who were not involved in participant enrolment or intervention delivery."

11b) If relevant, description of the similarity of interventions

(this item is usually not relevant for ehealth trials as it refers to similarity of a placebo or sham intervention to a active medication/intervention)

Does your paper address CONSORT subitem 11b? *

Copy and paste relevant sections from the manuscript (include quotes in quotation marks "like this" to indicate direct quotes from your manuscript), or elaborate on this item by providing additional information not in the ms, or briefly explain why the item is not applicable/relevant for your study

“Because of the nature of the intervention, participants could not be blinded to group assignment. However, outcome assessment was based on step count data automatically recorded by participants’ smartphones, which reduced the potential for assessor bias. Analyses were performed by investigators who were not involved in participant enrolment or intervention delivery.”

12a) Statistical methods used to compare groups for primary and secondary outcomes

NPT: When applicable, details of whether and how the clustering by care providers or centers was addressed

Does your paper address CONSORT subitem 12a? *

Copy and paste relevant sections from the manuscript (include quotes in quotation marks "like this" to indicate direct quotes from your manuscript), or elaborate on this item by providing additional information not in the ms, or briefly explain why the item is not applicable/relevant for your study

“For unadjusted analyses, changes in weekly average daily step counts from baseline were calculated for each study week by treatment arm.
For adjusted analyses, we used linear mixed models with restricted maximum likelihood estimation to account for the hierarchical structure of this cluster-randomized trial, with repeated measures nested within participants and participants nested within groups. Random intercepts were included at both participant and group levels. For the primary outcome—changes in weekly average daily step counts from baseline to week 12—step data were collected via smartphones, and baseline covariates were obtained through questionnaires. For adjusted analyses, we included baseline covariates that were selected a priori, before unblinding, based on theoretical relevance and prior studies of physical activity interventions in older adults. The adjusted model included operating system (Android/iOS), study field, age, gender, education level (high school or less vs. college or more), marital status, baseline smartphone use, self-rated health (0–10), total physical activity, and walking time (≥30 minutes/day). ”

“For secondary outcomes, linear mixed models were used for continuous variables (total physical activity and number of smartphone use purposes), and generalized linear mixed models with a logit link for binary outcomes (walking time and daily smartphone use). Adjustments were made using the same baseline covariates for total physical activity and walking time. Models for smartphone use and smartphone use purposes adjusted for operating system, study field, age, gender, education, marital status, and baseline smartphone use; the latter also included baseline smartphone use purposes.”

12a-i) Imputation techniques to deal with attrition / missing values

Imputation techniques to deal with attrition / missing values: Not all participants will use the intervention/comparator as intended and attrition is typically high in ehealth trials. Specify how participants who did not use the application or dropped out from the trial were treated in the statistical analysis (a complete case analysis is strongly discouraged, and simple imputation techniques such as LOCF may also be problematic [4]).

	1	2	3	4	5	
subitem not at all important	○	○	○	○	○	essential

Does your paper address subitem 12a-i? *

Copy and paste relevant sections from the manuscript (include quotes in quotation marks "like this" to indicate direct quotes from your manuscript), or elaborate on this item by providing additional information not in the ms, or briefly explain why the item is not applicable/relevant for your study

"Days with <1000 steps and other missing data were imputed, based on evidence these often reflect incomplete capture[24, 27]. Single imputation was performed using the missRanger package in R, with demographic, behavioral, and health variables included for both primary and secondary outcomes. Separate imputation models were created for step count data (n = 117) and questionnaire-based outcomes (n = 124)."

"Sensitivity analyses were conducted using (1) unimputed data, excluding days with missing or <1000 steps, (2) multiple imputation (10 imputations via random forest, with effect estimates combined using Rubin's rules); "

12b) Methods for additional analyses, such as subgroup analyses and adjusted analyses

Does your paper address CONSORT subitem 12b? *

Copy and paste relevant sections from the manuscript (include quotes in quotation marks "like this" to indicate direct quotes from your manuscript), or elaborate on this item by providing additional information not in the ms, or briefly explain why the item is not applicable/relevant for your study

"For unadjusted analyses, changes in weekly average daily step counts from baseline were calculated for each study week by treatment arm.
For adjusted analyses, we used linear mixed models with restricted maximum likelihood estimation to account for the hierarchical structure of this cluster-randomized trial, with repeated measures nested within participants and participants nested within groups. Random intercepts were included at both participant and group levels. For the primary outcome—changes in weekly average daily step counts from baseline to week 12—step data were collected via smartphones, and baseline covariates were obtained through questionnaires. For adjusted analyses, we included baseline covariates that were selected a priori, before unblinding, based on theoretical relevance and prior studies of physical activity interventions in older adults. The adjusted model included operating system (Android/iOS), study field, age, gender, education level (high school or less vs. college or more), marital status, baseline smartphone use, self-rated health (0–10), total physical activity, and walking time (≥30 minutes/day)."

"Days with <1000 steps and other missing data were imputed, based on evidence these often reflect incomplete capture[24, 27]. Single imputation was performed using the missRanger package in R, with demographic, behavioral, and health variables included for both primary and secondary outcomes. Separate imputation models were created for step count data (n = 117) and questionnaire-based outcomes (n = 124)."

"Sensitivity analyses were conducted using (1) unimputed data, excluding days with missing or <1000 steps, (2) multiple imputation (10 imputations via random forest, with effect estimates combined using Rubin's rules); "

"Because a baseline age imbalance between arms was observed, we conducted an post-hoc exploratory subgroup analysis by age. Participants were divided at the median age of 80 years, and separate adjusted mixed-effects models were fitted for each subgroup using the same covariates as the main model. In addition, we fitted an interaction model including a treatment × week × age term to formally test whether age modified the intervention effect."

X26) REB/IRB Approval and Ethical Considerations [recommended as subheading under "Methods"] (not a CONSORT item)

X26-i) Comment on ethics committee approval

	1	2	3	4	5	
subitem not at all important	○	○	○	○	○	essential

Does your paper address subitem X26-i?

Copy and paste relevant sections from the manuscript (include quotes in quotation marks "like this" to indicate direct quotes from your manuscript), or elaborate on this item by providing additional information not in the ms, or briefly explain why the item is not applicable/relevant for your study

“The trial received ethical approval from the Chiba University Ethical Committee (protocol: M10626) and was registered with the University Hospital Medical Information Network (UMIN000051904). Informed consent was obtained from all participants before randomization. All procedures were conducted in accordance with the Declaration of Helsinki.”

x26-ii) Outline informed consent procedures

Outline informed consent procedures e.g., if consent was obtained offline or online (how? Checkbox, etc.?), and what information was provided (see 4a-ii). See [6] for some items to be included in informed consent documents.

	1	2	3	4	5	
subitem not at all important	○	○	○	○	○	essential

Does your paper address subitem X26-ii?

Copy and paste relevant sections from the manuscript (include quotes in quotation marks "like this" to indicate direct quotes from your manuscript), or elaborate on this item by providing additional information not in the ms, or briefly explain why the item is not applicable/relevant for your study

“Informed consent was obtained from all participants before randomization. All procedures were conducted in accordance with the Declaration of Helsinki."

X26-iii) Safety and security procedures

Safety and security procedures, incl. privacy considerations, and any steps taken to reduce the likelihood or detection of harm (e.g., education and training, availability of a hotline)

	1	2	3	4	5	
subitem not at all important	○	○	○	○	○	essential

Does your paper address subitem X26-iii?

Copy and paste relevant sections from the manuscript (include quotes in quotation marks "like this" to indicate direct quotes from your manuscript), or elaborate on this item by providing additional information not in the ms, or briefly explain why the item is not applicable/relevant for your study

回答を入力

RESULTS

13a) For each group, the numbers of participants who were randomly assigned, received intended treatment, and were analysed for the primary outcome

NPT: The number of care providers or centers performing the intervention in each group and the number of patients treated by each care provider in each center

Does your paper address CONSORT subitem 13a? *

Copy and paste relevant sections from the manuscript (include quotes in quotation marks "like this" to indicate direct quotes from your manuscript), or elaborate on this item by providing additional information not in the ms, or briefly explain why the item is not applicable/relevant for your study

"At the interim analysis, 40 groups comprising 156 participants were allocated either to an intervention arm (n = 80, 20 groups) or a control arm (n = 76, 20 groups; see Figure 2). Of the 156 enrolled participants, 124 (79.5%) completed the 3-month follow-up evaluation (64 in the intervention group and 60 in the control group), with step-count data successfully linked for 117 participants."

13b) For each group, losses and exclusions after randomisation, together with reasons

Does your paper address CONSORT subitem 13b? (NOTE: Preferably, this is shown in a CONSORT flow diagram) *

Copy and paste relevant sections from the manuscript (include quotes in quotation marks "like this" to indicate direct quotes from your manuscript), or elaborate on this item by providing additional information not in the ms, or briefly explain why the item is not applicable/relevant for your study

“At the interim analysis, 40 groups comprising 156 participants were allocated either to an intervention arm (n = 80, 20 groups) or a control arm (n = 76, 20 groups; see Figure 2). Of the 156 enrolled participants, 124 (79.5%) completed the 3-month follow-up evaluation (64 in the intervention group and 60 in the control group), with step-count data successfully linked for 117 participants.”

13b-i) Attrition diagram

Strongly recommended: An attrition diagram (e.g., proportion of participants still logging in or using the intervention/comparator in each group plotted over time, similar to a survival curve) or other figures or tables demonstrating usage/dose/engagement.

	1	2	3	4	5	
subitem not at all important	○	○	○	○	○	essential

Does your paper address subitem 13b-i?

Copy and paste relevant sections from the manuscript or cite the figure number if applicable (include quotes in quotation marks "like this" to indicate direct quotes from your manuscript), or elaborate on this item by providing additional information not in the ms, or briefly explain why the item is not applicable/relevant for your study

回答を入力

14a) Dates defining the periods of recruitment and follow-up

Does your paper address CONSORT subitem 14a? *

Copy and paste relevant sections from the manuscript (include quotes in quotation marks "like this" to indicate direct quotes from your manuscript), or elaborate on this item by providing additional information not in the ms, or briefly explain why the item is not applicable/relevant for your study

From the Methods section:

“The study was conducted in Sumida Ward, Tokyo, Japan, from September to December 2023, and in Chiba City, Chiba, Japan, from June to September 2024.”

From the Participants section:

“Recruitment took place from September 2023 to June 2024, in partnership with the Sumida Ward local government and senior clubs in Chiba City.”

From the Results section:

“Of the 156 enrolled participants, 124 (79.5%) completed the 3-month follow-up evaluation (64 in the intervention group and 60 in the control group), with step-count data successfully linked for 117 participants.”

14a-i) Indicate if critical “secular events” fell into the study period

Indicate if critical “secular events” fell into the study period, e.g., significant changes in Internet resources available or “changes in computer hardware or Internet delivery resources”

	1	2	3	4	5	
subitem not at all important	○	○	○	○	○	essential

Does your paper address subitem 14a-i?

Copy and paste relevant sections from the manuscript (include quotes in quotation marks "like this" to indicate direct quotes from your manuscript), or elaborate on this item by providing additional information not in the ms, or briefly explain why the item is not applicable/relevant for your study

回答を入力

14b) Why the trial ended or was stopped (early)

Does your paper address CONSORT subitem 14b? *

Copy and paste relevant sections from the manuscript (include quotes in quotation marks "like this" to indicate direct quotes from your manuscript), or elaborate on this item by providing additional information not in the ms, or briefly explain why the item is not applicable/relevant for your study

“At the interim analysis, 40 groups comprising 156 participants were allocated either to an intervention arm (n = 80, 20 groups) or a control arm (n = 76, 20 groups; see Figure 2). Of the 156 enrolled participants, 124 (79.5%) completed the 3-month follow-up evaluation (64 in the intervention group and 60 in the control group), with step-count data successfully linked for 117 participants.”

15) A table showing baseline demographic and clinical characteristics for each group

NPT: When applicable, a description of care providers (case volume, qualification, expertise, etc.) and centers (volume) in each group

Does your paper address CONSORT subitem 15? *

Copy and paste relevant sections from the manuscript (include quotes in quotation marks "like this" to indicate direct quotes from your manuscript), or elaborate on this item by providing additional information not in the ms, or briefly explain why the item is not applicable/relevant for your study

“As shown in Table 1, baseline characteristics were generally well balanced between arms, except for a significant difference in mean age (p = 0.009). Mean (SD) baseline daily steps were 3975 (1590) for the control arm and 4318 (1513) for the intervention arm.”

15-i) Report demographics associated with digital divide issues

In ehealth trials it is particularly important to report demographics associated with digital divide issues, such as age, education, gender, social-economic status, computer/Internet/ehealth literacy of the participants, if known.

	1	2	3	4	5	
subitem not at all important	○	○	○	○	○	essential

Does your paper address subitem 15-i? *

Copy and paste relevant sections from the manuscript (include quotes in quotation marks "like this" to indicate direct quotes from your manuscript), or elaborate on this item by providing additional information not in the ms, or briefly explain why the item is not applicable/relevant for your study

We captured age, education, gender, smartphone ownership, and baseline smartphone use, which are key indicators of potential digital divide.
“For adjusted analyses, we included baseline covariates that were selected a priori… The adjusted model included operating system (Android/iOS), study field, age, gender, education level (high school or less vs. college or more), marital status, baseline smartphone use, self-rated health (0–10), total physical activity, and walking time (≥30 minutes/day).”

16) For each group, number of participants (denominator) included in each analysis and whether the analysis was by original assigned groups

16-i) Report multiple “denominators” and provide definitions

Report multiple “denominators” and provide definitions: Report N’s (and effect sizes) “across a range of study participation [and use] thresholds” [1], e.g., N exposed, N consented, N used more than x times, N used more than y weeks, N participants “used” the intervention/comparator at specific pre-defined time points of interest (in absolute and relative numbers per group). Always clearly define “use” of the intervention.

	1	2	3	4	5	
subitem not at all important	○	○	○	○	○	essential

Does your paper address subitem 16-i? *

Copy and paste relevant sections from the manuscript (include quotes in quotation marks "like this" to indicate direct quotes from your manuscript), or elaborate on this item by providing additional information not in the ms, or briefly explain why the item is not applicable/relevant for your study

From the Results section:

“At the interim analysis, 40 groups comprising 156 participants were allocated either to an intervention arm (n = 80, 20 groups) or a control arm (n = 76, 20 groups; see Figure 2). Of the 156 enrolled participants, 124 (79.5%) completed the 3-month follow-up evaluation (64 in the intervention group and 60 in the control group), with step-count data successfully linked for 117 participants. ”

16-ii) Primary analysis should be intent-to-treat

Primary analysis should be intent-to-treat, secondary analyses could include comparing only “users”, with the appropriate caveats that this is no longer a randomized sample (see 18-i).

	1	2	3	4	5	
subitem not at all important	○	○	○	○	○	essential

Does your paper address subitem 16-ii?

Copy and paste relevant sections from the manuscript (include quotes in quotation marks "like this" to indicate direct quotes from your manuscript), or elaborate on this item by providing additional information not in the ms, or briefly explain why the item is not applicable/relevant for your study

回答を入力

17a) For each primary and secondary outcome, results for each group, and the estimated effect size and its precision (such as 95% confidence interval)

Does your paper address CONSORT subitem 17a? *

Copy and paste relevant sections from the manuscript (include quotes in quotation marks "like this" to indicate direct quotes from your manuscript), or elaborate on this item by providing additional information not in the ms, or briefly explain why the item is not applicable/relevant for your study

From the Results (Primary outcome) section:

“The primary outcome was the change in average daily steps at week 12 compared with baseline. In the fully adjusted mixed-effects model, the intervention arm recorded significantly higher steps than the control arm at week 12 (difference = 479 steps; 95% CI = 41 to 918; p = 0.03) (Table 2). ”

From the Results (Secondary outcomes) section:

“Exploratory analyses of weekly step counts (weeks 1–11) showed that the intervention arm had higher steps at week 1 and week 9 (borderline significance; p = 0.08 and 0.07, respectively) and significantly higher steps at week 11 (difference = 509 steps; 95% CI = 70 to 947; p = 0.02) (Table 2).
In a further exploratory analysis aggregating daily steps by month, the intervention arm outperformed the control arm by an estimated 371 steps (95% CI = 0 to 742; p = 0.05) at month 3, although months 1 and 2 did not show significant differences (Supplementary Table 3).
No significant group differences were observed for physical activity measured by total METs or for self-reported walking time (≥30 minutes/day). The adjusted difference in total METs was 714 MET-minutes/week (95% CI = −204 to 1631; p = 0.12), and the odds ratio for walking ≥30 minutes/day was 1.15 (95% CI = 0.42 to 3.20; p = 0.78) (Supplementary Table 4). However, there was a suggestive trend toward an increase in daily smartphone use among participants in the intervention group, with an odds ratio of 3.8 (95% CI = 0.85 to 17.0; p = 0.08). In addition, the number of smartphone use purposes significantly increased among intervention participants compared to controls, with an adjusted mean difference of 0.59 (95% CI = 0.10 to 1.08; p = 0.02).”

17a-i) Presentation of process outcomes such as metrics of use and intensity of use

In addition to primary/secondary (clinical) outcomes, the presentation of process outcomes such as metrics of use and intensity of use (dose, exposure) and their operational definitions is critical. This does not only refer to metrics of attrition (13-b) (often a binary variable), but also to more continuous exposure metrics such as “average session length”. These must be accompanied by a technical description how a metric like a “session” is defined (e.g., timeout after idle time) [1] (report under item 6a).

	1	2	3	4	5	
subitem not at all important	○	○	○	○	○	essential

Does your paper address subitem 17a-i?

Copy and paste relevant sections from the manuscript (include quotes in quotation marks "like this" to indicate direct quotes from your manuscript), or elaborate on this item by providing additional information not in the ms, or briefly explain why the item is not applicable/relevant for your study

回答を入力

17b) For binary outcomes, presentation of both absolute and relative effect sizes is recommended

Does your paper address CONSORT subitem 17b? *

Copy and paste relevant sections from the manuscript (include quotes in quotation marks "like this" to indicate direct quotes from your manuscript), or elaborate on this item by providing additional information not in the ms, or briefly explain why the item is not applicable/relevant for your study

Our binary outcomes (walking ≥30 minutes/day, daily smartphone use) were secondary endpoints. We reported relative effect sizes (odds ratios with 95% CIs), but absolute effect sizes were not presented, consistent with the secondary nature of these outcomes.

18) Results of any other analyses performed, including subgroup analyses and adjusted analyses, distinguishing pre-specified from exploratory

Does your paper address CONSORT subitem 18? *

Copy and paste relevant sections from the manuscript (include quotes in quotation marks "like this" to indicate direct quotes from your manuscript), or elaborate on this item by providing additional information not in the ms, or briefly explain why the item is not applicable/relevant for your study

Exploratory post-hoc analyses
In exploratory subgroup analyses stratified by the median age of 80 years (younger: n = 57; older: n = 58), baseline age was well balanced within subgroups (younger: mean 76.2 vs. 76.3 years; older: mean 84.2 vs. 83.1 years). Intervention effects varied by age. Among older participants (≥80 years; n = 58), significant treatment effects were observed from weeks 3 through 12, with between-group differences exceeding 1,000 steps at multiple time points. In contrast, younger participants (<80 years; n = 57) showed inconsistent and occasionally negative effects, with no sustained significant benefits. Consistent with these findings, the treatment × week × age interaction model indicated that intervention effects became stronger among participants aged 80 years or older in later weeks. Detailed estimates from the subgroup and interaction analyses are provided in Supplementary Table 5.

18-i) Subgroup analysis of comparing only users

A subgroup analysis of comparing only users is not uncommon in ehealth trials, but if done, it must be stressed that this is a self-selected sample and no longer an unbiased sample from a randomized trial (see 16-iii).

	1	2	3	4	5	
subitem not at all important	○	○	○	○	○	essential

Does your paper address subitem 18-i?

Copy and paste relevant sections from the manuscript (include quotes in quotation marks "like this" to indicate direct quotes from your manuscript), or elaborate on this item by providing additional information not in the ms, or briefly explain why the item is not applicable/relevant for your study

回答を入力

19) All important harms or unintended effects in each group

(for specific guidance see CONSORT for harms)

Does your paper address CONSORT subitem 19? *

Copy and paste relevant sections from the manuscript (include quotes in quotation marks "like this" to indicate direct quotes from your manuscript), or elaborate on this item by providing additional information not in the ms, or briefly explain why the item is not applicable/relevant for your study

No important harms or unintended effects were detected.

19-i) Include privacy breaches, technical problems

Include privacy breaches, technical problems. This does not only include physical "harm" to participants, but also incidents such as perceived or real privacy breaches [1], technical problems, and other unexpected/unintended incidents. "Unintended effects" also includes unintended positive effects [2].

	1	2	3	4	5	
subitem not at all important	○	○	○	○	○	essential

Does your paper address subitem 19-i?

Copy and paste relevant sections from the manuscript (include quotes in quotation marks "like this" to indicate direct quotes from your manuscript), or elaborate on this item by providing additional information not in the ms, or briefly explain why the item is not applicable/relevant for your study

回答を入力

19-ii) Include qualitative feedback from participants or observations from staff/researchers

Include qualitative feedback from participants or observations from staff/researchers, if available, on strengths and shortcomings of the application, especially if they point to unintended/unexpected effects or uses. This includes (if available) reasons for why people did or did not use the application as intended by the developers.

	1	2	3	4	5	
subitem not at all important	○	○	○	○	○	essential

Does your paper address subitem 19-ii?

Copy and paste relevant sections from the manuscript (include quotes in quotation marks "like this" to indicate direct quotes from your manuscript), or elaborate on this item by providing additional information not in the ms, or briefly explain why the item is not applicable/relevant for your study

回答を入力

DISCUSSION

22) Interpretation consistent with results, balancing benefits and harms, and considering other relevant evidence

NPT: In addition, take into account the choice of the comparator, lack of or partial blinding, and unequal expertise of care providers or centers in each group

22-i) Restate study questions and summarize the answers suggested by the data, starting with primary outcomes and process outcomes (use)

Restate study questions and summarize the answers suggested by the data, starting with primary outcomes and process outcomes (use).

	1	2	3	4	5	
subitem not at all important	○	○	○	○	○	essential

Does your paper address subitem 22-i? *

Copy and paste relevant sections from the manuscript (include quotes in quotation marks "like this" to indicate direct quotes from your manuscript), or elaborate on this item by providing additional information not in the ms, or briefly explain why the item is not applicable/relevant for your study

"Short Summary

This cluster-randomized controlled trial demonstrates that a smartphone lecture program, combined with a mobile app-based peer support component, may produce modest but meaningful increases in step counts among older adults in Japan compared to a conventional smartphone lecture program. The intervention group recorded a statistically significant increase in average daily steps at week 12 (the pre-specified primary endpoint), with supportive differences observed at week 11 and borderline differences at weeks 1 and 9. All other weekly comparisons and monthly averages were exploratory and should be interpreted with caution, as no adjustment for multiple testing was applied. In addition, smartphone use and purposes were greater in the intervention arm. These results suggest that integrating peer support features into smartphone-based health initiatives could be a promising strategy for sustaining engagement and encouraging physical activity and smartphone use over time."

22-ii) Highlight unanswered new questions, suggest future research

Highlight unanswered new questions, suggest future research.

	1	2	3	4	5	
subitem not at all important	○	○	○	○	○	essential

Does your paper address subitem 22-ii?

Copy and paste relevant sections from the manuscript (include quotes in quotation marks "like this" to indicate direct quotes from your manuscript), or elaborate on this item by providing additional information not in the ms, or briefly explain why the item is not applicable/relevant for your study

回答を入力

20) Trial limitations, addressing sources of potential bias, imprecision, and, if relevant, multiplicity of analyses

20-i) Typical limitations in ehealth trials

Typical limitations in ehealth trials: Participants in ehealth trials are rarely blinded. Ehealth trials often look at a multiplicity of outcomes, increasing risk for a Type I error. Discuss biases due to non-use of the intervention/usability issues, biases through informed consent procedures, unexpected events.

	1	2	3	4	5	
subitem not at all important	○	○	○	○	○	essential

Does your paper address subitem 20-i? *

Copy and paste relevant sections from the manuscript (include quotes in quotation marks "like this" to indicate direct quotes from your manuscript), or elaborate on this item by providing additional information not in the ms, or briefly explain why the item is not applicable/relevant for your study

Blinding
Because of the nature of the intervention, participants and local staff could not be blinded to group assignment. However, outcome assessment was based on step count data automatically recorded by participants' smartphones, and data analysts were blinded to allocation, reducing the risk of bias.
The two study arms differed in that only the intervention group received access to the MinChalle app and related training. Therefore, the interventions could not be made similar in appearance or content.

21) Generalisability (external validity, applicability) of the trial findings

NPT: External validity of the trial findings according to the intervention, comparators, patients, and care providers or centers involved in the trial

21-i) Generalizability to other populations

Generalizability to other populations: In particular, discuss generalizability to a general Internet population, outside of a RCT setting, and general patient population, including applicability of the study results for other organizations

	1	2	3	4	5	
subitem not at all important	○	○	○	○	○	essential

Does your paper address subitem 21-i?

Copy and paste relevant sections from the manuscript (include quotes in quotation marks "like this" to indicate direct quotes from your manuscript), or elaborate on this item by providing additional information not in the ms, or briefly explain why the item is not applicable/relevant for your study

Finally, the external validity of our findings should be interpreted with caution. This trial was conducted in two urban areas of Japan, where walkable environments and access to digital infrastructure are relatively well developed. The results may not directly translate to rural areas with limited walkability or to contexts with lower smartphone and internet penetration. In addition, cultural differences in peer support and technology use may influence the effectiveness of similar interventions in international settings. These contextual factors should be carefully considered when applying our findings to broader populations.

21-ii) Discuss if there were elements in the RCT that would be different in a routine application setting

Discuss if there were elements in the RCT that would be different in a routine application setting (e.g., prompts/reminders, more human involvement, training sessions or other co-interventions) and what impact the omission of these elements could have on use, adoption, or outcomes if the intervention is applied outside of a RCT setting.

	1	2	3	4	5	
subitem not at all important	○	○	○	○	○	essential

Does your paper address subitem 21-ii?

Copy and paste relevant sections from the manuscript (include quotes in quotation marks "like this" to indicate direct quotes from your manuscript), or elaborate on this item by providing additional information not in the ms, or briefly explain why the item is not applicable/relevant for your study

回答を入力

OTHER INFORMATION

23) Registration number and name of trial registry

Does your paper address CONSORT subitem 23? *

Copy and paste relevant sections from the manuscript (include quotes in quotation marks "like this" to indicate direct quotes from your manuscript), or elaborate on this item by providing additional information not in the ms, or briefly explain why the item is not applicable/relevant for your study

”The trial received ethical approval from the Chiba University Ethical Committee (protocol: M10626) and was registered with the University Hospital Medical Information Network (UMIN000051904).”

24) Where the full trial protocol can be accessed, if available

Does your paper address CONSORT subitem 24? *

Cite a Multimedia Appendix, other reference, or copy and paste relevant sections from the manuscript (include quotes in quotation marks "like this" to indicate direct quotes from your manuscript), or elaborate on this item by providing additional information not in the ms, or briefly explain why the item is not applicable/relevant for your study

The full trial protocol is not available.

25) Sources of funding and other support (such as supply of drugs), role of funders

Does your paper address CONSORT subitem 25? *

Copy and paste relevant sections from the manuscript (include quotes in quotation marks "like this" to indicate direct quotes from your manuscript), or elaborate on this item by providing additional information not in the ms, or briefly explain why the item is not applicable/relevant for your study

Funding Statement
This study was funded by A10 Lab Inc. Atsushi Nakagomi and Katsunori Kondo have received research funding from A10 Lab Inc. to evaluate the MinChalle app and provide feedback on service improvement based on study findings. The sponsor's role was limited to introducing municipalities for recruitment and providing raw step count data. The sponsor had no involvement in the study design, data analysis, data interpretation, manuscript writing, or the decision to submit the article for publication. To safeguard independence, all statistical analyses were conducted solely by academic investigators (NA and AN), with independent oversight by YK.

X27) Conflicts of Interest (not a CONSORT item)

X27-i) State the relation of the study team towards the system being evaluated

In addition to the usual declaration of interests (financial or otherwise), also state the relation of the study team towards the system being evaluated, i.e., state if the authors/evaluators are distinct from or identical with the developers/sponsors of the intervention.

	1	2	3	4	5	
subitem not at all important	○	○	○	○	○	essential

Does your paper address subitem X27-i?

Copy and paste relevant sections from the manuscript (include quotes in quotation marks "like this" to indicate direct quotes from your manuscript), or elaborate on this item by providing additional information not in the ms, or briefly explain why the item is not applicable/relevant for your study

回答を入力

About the CONSORT EHEALTH checklist

As a result of using this checklist, did you make changes in your manuscript? *

- (•) yes, major changes
- ( ) yes, minor changes
- ( ) no

What were the most important changes you made as a result of using this checklist?

回答を入力

How much time did you spend on going through the checklist INCLUDING making changes in your manuscript *

We spent on going through the checklist for five hours.

As a result of using this checklist, do you think your manuscript has improved? *

- ( ) yes
- ( ) no
- ( ) その他:

Would you like to become involved in the CONSORT EHEALTH group?

This would involve for example becoming involved in participating in a workshop and writing an "Explanation and Elaboration" document

- yes
- no
- その他:

Any other comments or questions on CONSORT EHEALTH

回答を入力

STOP - Save this form as PDF before you click submit

To generate a record that you filled in this form, we recommend to generate a PDF of this page (on a Mac, simply select "print" and then select "print as PDF") before you submit it.

When you submit your (revised) paper to JMIR, please upload the PDF as supplementary file.

Don't worry if some text in the textboxes is cut off, as we still have the complete information in our database. Thank you!

Final step: Click submit !

Click submit so we have your answers in our database!

送信 フォームをクリア

Google フォームでパスワードを送信しないでください。

このコンテンツは Google が作成または承認したものではありません。 - フォームのオーナーに問い合わせる - 利用規約 - プライバシー ポリシー

このフォームが不審だと思われる場合 報告

Google フォーム
